# Fabrication of Reactive Poly(Phenyl-Substituted Siloxanes/Silsesquioxanes) with Si‒H and Alkoxy Functional Groups via the Piers–Rubinsztajn Reaction

**DOI:** 10.3390/polym10091006

**Published:** 2018-09-10

**Authors:** Minghao Yi, Xunjun Chen, Shufang Wu, Jianfang Ge, Xinhua Zhou, Guoqiang Yin

**Affiliations:** 1 Engineering Research Center of Silicone Electronic Fine Chemicals, Zhongkai University of Agriculture and Engineering, Guangzhou 510220, China; MHYi0848@163.com (M.Y.); sfwu2018@163.com (S.W.); ge650704@163.com (J.G.); cexinhuazhou@163.com (X.Z.); 2 Guangzhou Key Laboratory for Efficient Utilization of Agricultural Chemicals, Zhongkai University of Agriculture and Engineering, Guangzhou 510220, China; yingq007@163.com

**Keywords:** poly(phenyl-substituted siloxanes/silsesquioxanes), Piers–Rubinsztajn reaction, molecular bridges, functional groups, light-emitting diode (LED) packaging industry

## Abstract

Poly(phenyl-substituted siloxanes/silsesquioxanes) are obtained by the Piers–Rubinsztajn (PR) reaction of hydrogen-containing siloxanes (HCS) with diphenyldialkoxysilanes such as diphenyldimethoxysilane and diphenyldiethoxysilane catalyzed by tris(pentafluorophenyl)borane. ^29^Si nuclear magnetic resonance (NMR) spectroscopy, gel permeation chromatography, and refractive index analysis revealed that apart from phenyl substituents and complex structures such as molecular bridges composed of D_2_^Ph2^[(C_6_H_5_)_2_Si(OSi)_2_], structures also existed in these polymers, having high refractive indexes (above 1.50) and high molecular weights (75.60 KDa·mol^−1^). As revealed by thermogravimetric analysis, these polymers have high thermal stability as well, with temperature at 5% mass loss (*T*_5%_) increasing by 182.5 °C and *R*_w_ (residual weight ratio) increasing by 5.17 times from 14.63% to 75.60%, as compared to HCS, exhibiting its potential application as resins for resisting strong heat. Such high-refractive-index and temperature-resistant poly(phenyl-substituted siloxanes/silsesquioxanes) with Si–H and alkoxy functional groups can be used as a good addition-type crosslinking agent with adhesion-promoting properties or a special curing agent that can solidify silicone materials through simultaneous addition and condensation reactions, which has potential application in the light-emitting diode (LED) packaging industry.

## 1. Introduction

Even though its synthesis was first reported in the early 1960s, with no obvious purpose, tris(pentafluorophenyl)borane (B(C_6_F_5_)_3_) remained uninvestigated for decades [1,2]. However, Marks and his co-workers as well as Piers eventually reported B(C_6_F_5_)_3_ as an excellent polymerization catalyst in the 1990s [3,4,5,6]. B(C_6_F_5_)_3_ readily catalyzes the transfer of hydride ions from silicon to organic compounds. Thus, it is not only used as a catalyst in the reduction of organic compounds by hydrosilanes [7,8]. but also in hydrosilylation reactions in organosilicon chemistry, leading to the formation of siloxane or silylether bonds [9,10]. Typical oxygen-based nucleophiles containing –OH or alkoxy groups [11] including phenols [11] and even graphene oxide [12] and lignin [13,14] are effectively reduced by hydrosilanes in the presence of B(C_6_F_5_)_3_ to give alkanes or silyl ethers—this is the Piers–Rubinsztajn (PR) reaction, which is a kind of hydrosilylation reaction.

A convenient synthetic method to more well-defined polysiloxanes is the dehydrogenative coupling of organohydrosilanes with organohydrosilanols owing to the high selectivity and easy removal of the hydrogen by-product [15]. However, this type of reaction requires relatively high concentrations (≥0.1 mol %) of precious metal catalysts such as platinum, palladium, ruthenium, or rhodium. Furthermore, only moderate molecular-weight polymers are achieved, and undesired silanol self-condensation can occur and lead to the disruption of a perfectly alternating polymer structure [16,17]. Because of economic concerns (platinum catalyst is expensive) and environmental concerns (tin catalysts are currently under scrutiny by regulatory agencies in many parts of the world [18]), replacing metals in moderate and efficient polymerization processes is desirable. In sharp contrast, hydrosilylation reactions catalyzed by B(C_6_F_5_)_3_, the “metal-free” catalyst [19], can also be used to synthesize well-defined polysiloxanes [20]. These reactions are often carried out under stringently controlled conditions to avoid the presence of water that will cause both structural defects and undesirable side reactions [21,22]. In addition, these reactions offer several advantages including high reaction effectiveness, convenient removal of (gaseous) by-products, mild temperatures, low catalyst concentrations, and the simple control of 3D structures by the opportune use of simple starting materials. Moreover, complex structures can be prepared that are otherwise unavailable by traditional routes [23].

The traditional route to preparing phenyl-substituted polysiloxanes is the hydrolytic polymerization of chlorosilane. The process causes strong acid contamination and produces silanol as a by-product, which leads to a decrease in the degree of crosslinking and interferes with the polymer structure.

Herein, we used B(C_6_F_5_)_3_ to catalyze the dehydrocarbon polycondensation, i.e., PR reaction under anhydrous conditions, of diphenyldialkoxysilanes with hydrogen-containing siloxanes (HCS) to yield specially designed poly(phenyl-substituted siloxanes/silsesquioxanes) along with lower alkanes as inert by-products at very low catalyst concentrations. The polymers possess good thermal stability and satisfactory refractive index because of the presence of phenyl substituents and molecular bridges [24]. In addition, the polymer retains reactive functional groups such as the Si‒H bonds and alkoxy groups, which would render it suitable as an additive crosslinking agent or a special curing agent.

## 2. Materials and Methods

### 2.1. Materials

HCS (content of hydrogen: 1.6%, purity > 99.5%) were obtained from Dongguan LetterKang Silicone Co., Ltd. (Dongguan, China), and toluene was obtained from Guangzhou YiMa Science and Technology Co., Ltd. (Guangzhou, China). B(C_6_F_5_)_3_ (purity > 98%) was purchased from Jilin University (Changchun, China). Diphenyldiethoxysilane (DPDES, purity > 99%) was supplied by Shanghai XinYu New Materials and Technology Co., Ltd. (Shanghai, China), and diphenyldimethoxysilane (DPDMS, purity > 99%) was provided by Guangzhou ShuangTao Fine Chemical Co., Ltd. (Guangzhou, China).

### 2.2. Dehydrocarbon Polycondensation of Diphenyldimethoxysilane (DPDMS) with Hydrogen-Containing Siloxanes (HCS)

The molar ratio of the alkoxy groups in DPDMS to the Si‒H Bonds in HCS was set at 0.8. Dry toluene (60.95 g), B(C_6_F_5_)_3_, (55.16 mg, 107.7 µmol) and HCS (17 g, molar amount of Si-H bond: 272.0 mmol) were loaded into a 250 mL three-necked round bottom flask equipped with a thermometer, reflux condenser, and magnetic stirrer. DPDMS (26.59 g, 108.8 mmol) was dissolved in toluene (20 g), and then added dropwise to the above solution for 3 h by a syringe pump at the required temperature. To obtain the product, the reaction must proceed for 1 h. After completion of the reaction, 2 g of activated carbon was introduced into the system to adsorb the catalyst. Thereafter, adsorption and suction filtration were performed twice after stirring for 20 min. Finally, the solvent and the product were separated by reduced-pressure distillation at 150 °C for 30 min by inletting air when the vacuum was below −0.095 MPa. The detailed experimental conditions and results are shown in Table 1.

### 2.3. Dehydrocarbon Polycondensation of Diphenyldiethoxysilane (DPDES) with HCS

The molar ratio of the alkoxy groups in DPDES to the Si‒H bonds in HCS was set at 0.8. Dry toluene (66.92 g), B(C_6_F_5_)_3_,(59.53 mg, 116.3 µmol) and HCS (17 g·mol amount of Si–H bond: 272.0 mmol) were loaded into a 250 mL three-necked round bottom flask equipped with a thermometer, reflux condenser, and magnetic stirrer. DPDES (29.64 g, 108.8 mmol) was dissolved in toluene (20 g), and then added dropwise to the above solution for 3 h by a syringe pump at the required temperature. To obtain the product, the reaction must proceed for 1 h. After completion of the reaction, 2 g of activated carbon was introduced into the system to adsorb the catalyst. Thereafter, adsorption and suction filtration was performed twice after stirring for 20 min. Finally, the solvent and the product were separated by reduced-pressure distillation at 150 °C for 30 min by inletting air when the vacuum was below −0.095 MPa. The detailed experimental conditions and results are shown in Table 1.

### 2.4. Measurements

The geometries of the various reactants and intermediates are optimized at the B3LYP/6-311G or B3LYP/STO-3G level based on the quantum chemical density functional theory (DFT). The refractive indexes were measured using a WZS-1 refractometer (Shanghai Optical Instrument Factory, Shanghai, China) at 25 °C. ^1^H nuclear magnetic resonance (NMR) (400 MHz, CDCl_3_, TMS) and ^29^Si NMR (400 MHz, CDCl_3_) spectra were recorded using a Bruker AVANCE AV 400 MHz spectrometer (Bruker Co., Karlsruhe, Germany) at room temperature. Gel permeation chromatography (GPC) was carried out on a Waters 1525/2414 chromatograph (WATERS Co., Milford, MA, USA) in a linear column eluted with tetrahydrofuran at a flow rate of 1.0 mL·min^−1^. Thermogravimetric analysis (TGA) was carried out on a TGA 2 thermogravimetric analyzer (Mettler-Toledo AG Co., Columbus, OH, USA) in nitrogen atmosphere (at a flow rate of 20 mL·min^−1^) from 40 to 700 °C at a heating rate of 10 °C·min^−1^.

## 3. Results and Discussion

As shown in Table 2, because of the strong electron-withdrawing effect of the ‒(C_6_F_5_) groups in B(C_6_F_5_)_3_, the electron density of the central B atom decreases (q_B_ = 0.802). This is in line with the behaviour of B(C_6_F_5_)_3_ like a Lewis acid [25]. When only miniHCS (((CH_3_)_3_SiOSiH_2_O)_2_, simplified model of HCS) is present, the difference in negative charge distribution on the active hydrogen is in the range of 0.0012–0.0072 Å, and the difference in the Si‒H bond length is only 0.0001–0.0008 Å. When B(C_6_F_5_)_3_ and miniHCS coexist, the positive charge of the B atom decreases by 0.5275, and the negative charge of H(38) in miniHCS, which may be closest to B(C_6_F_5_)_3_, is 0.0188–1.0353 higher than that of the other active hydrogens. At the same time, because of the coulomb force between the B atom and the H(38) atom, the H(38)‒Si(36) bond extended by 0.0054‒0.0072 Å compared with the other active Si‒H bonds in miniHCS, which implies that the difference increased by 6.75‒72 times. This provides evidence that the Si‒H bonds activated gradually but not simultaneously; thus, there will be mono- and di-substituted stages in the phenyl substitution process.

**Scheme 1:**
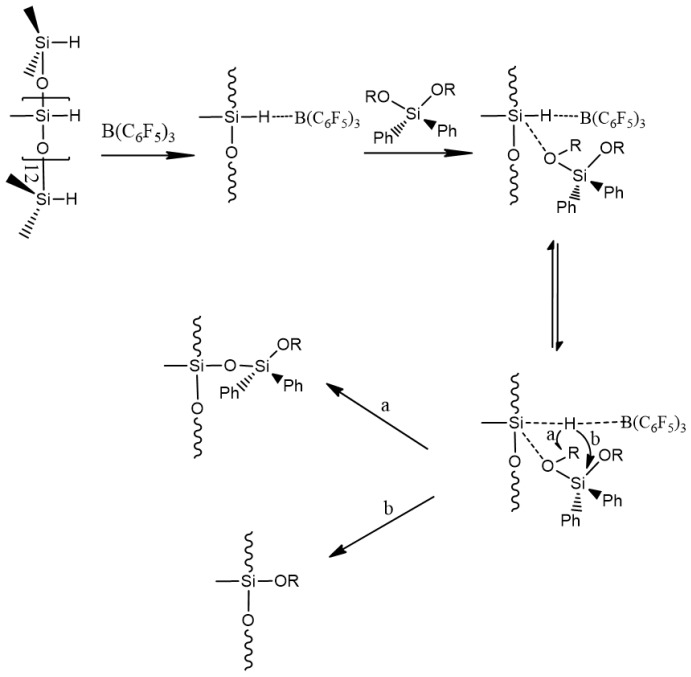
(**a**) Reaction between HCS and diphenyldialkoxysilane, (**b**) Functional group exchange reaction.

**Scheme 2:**
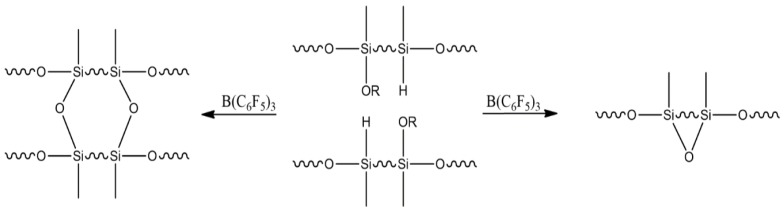
Formation of molecular bridges through condensation of functional groups on linear polymers. (R represents methyl or ethyl).

In systems where HCS coexist with B(C_6_F_5_)_3_, it is generally accepted that B(C_6_F_5_)_3_ first activates the Si‒H bonds and that the electron-rich O atoms in diphenyldialkoxysilane attack the Si positive atoms in the Si‒H‒B(C_6_F_5_)_3_ complex, eventually forming a stable structure with Si‒O‒Si bonds, accompanying the transfer of H atoms by breaking the Si‒H bonds and releasing alkanes [26]. In this process, the negative charges on the O atoms and even a slight difference in the Si‒O bond length in diphenyldialkoxysilane will greatly affect the PR reaction. The positive charge on the Si atom in DPDES (q_Si(1)_ = 1.1188) is 0.3289 higher than that on the Si atom in DPDMS (q_Si(1)_ = 0.7899), while the negative charge on the O atom in DPDMS (q_O(24)_ = −0.4336) is 0.0545‒0.0547 higher than that on the O atom in DPDES (q_O(24)_ = −0.3791, q_O(25)_ = −0.3789). In order to share fewer electron clouds, Si atoms and O atoms in DPDES should be close to each other. Because of this, the Si‒O bonds in DPDES are shorter than those in DPDMS (DPDES: R_O-Si_ = 1.6628 Å, DPDMS: R_O-Si_ = 1.666 Å). Of course, DPDMS with more negative charges on the O atoms, more electropositive C atoms (DPDMS: q_C(26)_ = 0.2844, DPDES: q_C(26)_ = 0.2034), and longer Si‒O bonds are more likely to be catalyzed by B(C_6_F_5_)_3_ to participate in the reaction. Furthermore, according to literature, the H group attacks the Si atom on the alkoxy group to promote an exchange reaction between the functional groups, which proceeds simultaneously with the PR reaction [9]. The calculation results of various compounds in Table 2 predict that the reaction system with DPDMS precursor is much easier and the degree of polymerization will be greater. Based on DFT, Scheme 1 reveals two reaction pathways that occur in this experiment. The polycondensation reaction is necessary to obtain high molecular-weight poly(phenyl-substituted siloxanes/silsesquioxanes), and the functional group exchange reaction can introduce alkoxy groups on the main chain of the polymers. To prevent the intermolecular polymerization of the byproducts ((C_6_H_5_)_2_SiHOR) produced by the exchange reaction, it must be ensured that the feed flow rate is slow enough to allow their polymerization with HCS. However, a part of the introduced alkoxy groups, owing to their propensity toward exchange reaction, has the potential to form macromolecular bridges (as shown in Scheme 2).

**Scheme 3:**
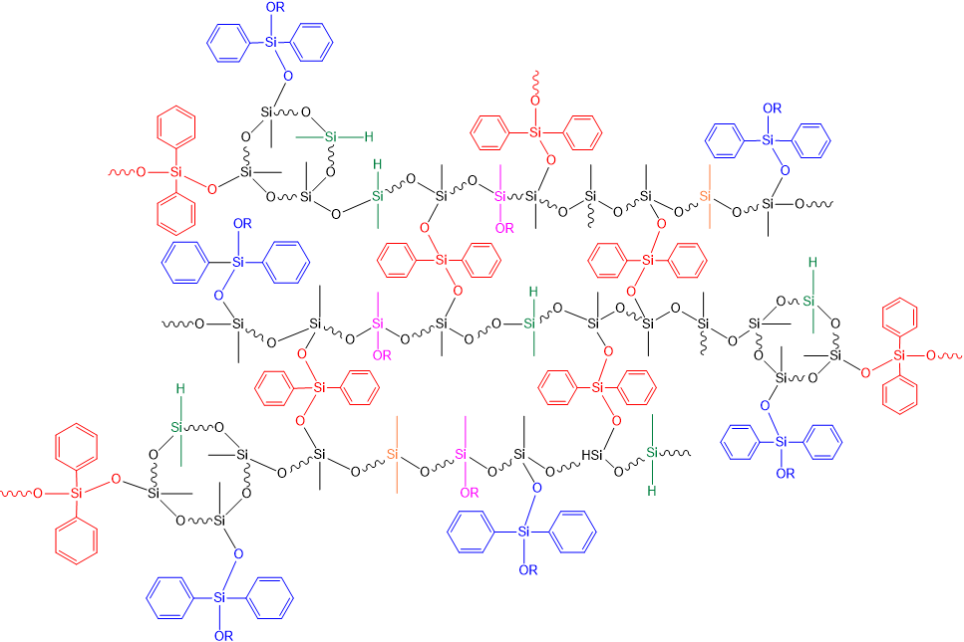
Polymers with complex structures obtained by condensation polymerization of diphenyldialkoxysilane and HCS. ‒OR represents ‒OCH_3_ or ‒OCH_2_CH_3._

Further, the reaction results in Table 1 indicate that the molecular weights of the polymers resulted from DPDMS and DPDES showed very good regularity. The higher the catalyst concentration, the greater is the molecular weight of the product and the broader is the molecular weight distribution. Polycondensation of alkoxysilanes with hydrosilanes is a strongly exothermic reaction [27] which is favored by low temperatures. Furthermore, the two groups with the highest molecular weight in the low-temperature range (0 °C, 10 °C) indicated that diphenyl structures were likely to form bridges between the macromolecules. We speculate that the difference in the molecular weights of the products may be related to different crosslinking degrees and complicated structures which are not limited to the planar linear structures and have many molecular bridges to form complex-space polymers (as shown in Scheme 3). Because silicone polymers are flexible owing to the rotational freedom of the Si‒O‒Si bonds, which may facilitate more Si-O-Si chain units except for diphenyl siloxy bridges between linear polymers [28]. In the two sets of experiments with different precursors, a large amount of catalyst always yields a high-molecular weight product with high polydispersity. At higher temperatures, the molecular weights of the obtained products decrease. For this PR reaction, increasing the concentration of reactants in toluene results in an increase in the molecular weight, the broadening of polydispersity, and an earlier gelation point. For the same reaction conditions, the molecular weights of the products obtained from DPDMS are slightly higher than those of the products obtained from DPDES. Together with the DFT calculation results, it was concluded that this is because the electron-rich O atoms and the more electropositive C atoms in the methoxy groups of DPDMS are more susceptible to the electron-deficient center of B(C_6_F_5_)_3_, which catalyzes this reaction.

**Figure 1:**
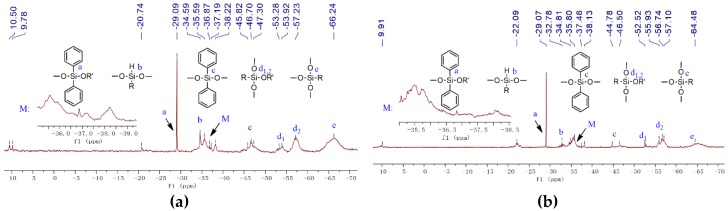

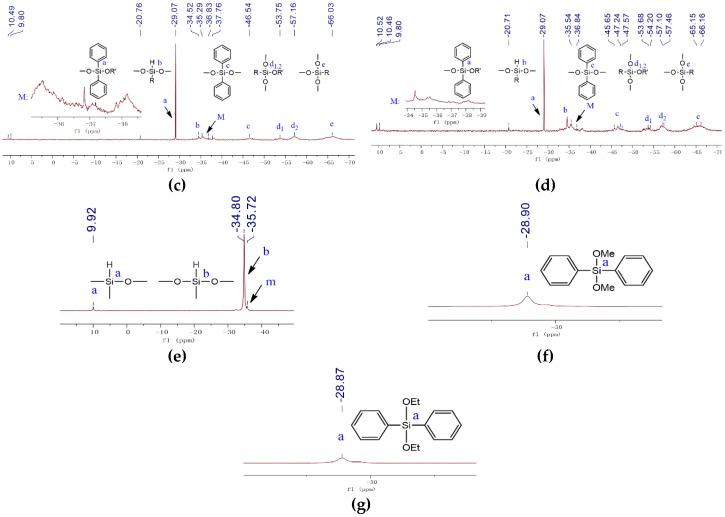
^29^Si nuclear magnetic resonance (NMR) spectra of D01 (**a**), D04 (**b**), D08 (**c**), D11 (**d**), HCS (**e**), DPDMS (**f**), and DPDES (**g**). M represents overclose molecular bridges. m represents small ring structure.

The ^29^Si NMR spectra of these polymers revealed that all the predicted structures were observed. The phenyl substitution process begins with the formation of a D_1_^Ph2^[(C_6_H_5_)_2_Si(OSi)OR] structure, followed by further polycondensation to form the D_2_^Ph2^[(C_6_H_5_)_2_Si(OSi)_2_] one. These reactions consume Si‒H bonds and form linear, annular, and networked structures. This implies that mono- and di-substituted structures would exist at the same time [29] because of the wrapped Si‒H bonds, the increase in steric hindrance due to the increase in the phenyl content of the polymer, and structural complication. Subsequently, the tendency of the polycondensation reaction starts to weaken due to the increase in the degree of polymerization and the reduction of hydrogen groups. The diphenyldialkoxysilane generates a Si atom signal from −28.87 to −28.90 ppm, while the polymer generates a signal at −29 ppm that can only arise from mono-substituted structures; this proves that part of the substitution process remained in the D_1_^Ph2^ structure without evolving into the D_2_^Ph2^ structure. Further polycondensation of the T_2_[R’OSi(OSi)_2_R] links is another way to form a bridge between the macromolecules; this explains the formation of a polymer such as D08 with a molecular weight of tens of thousands with few D_2_^Ph2^[(C_6_H_5_)_2_Si(OSi)_2_] structures in some cases. Indeed, ^29^Si NMR spectroscopy can be used to confirm the appearance of macrocyclic structures because the signal generated from the Si in the ring generally has a lower field than the Si atoms of the unconstrained linear polymer with similar structures do [30]. The HCS spectrum shows the presence of small ring structures (m in Figure 1e) although HCS was obtained by ring-opening synthesis. Similar signals of suspected macrocyclic structures (M in Figure 1a–d) are more pronounced and more numerous in the polymers. This may be due to the fact that some molecular bridges are too close together and result in higher strain energy, causing signals of similar macrocyclic structures [31]. However, we have no effective tools to verify the presence of macrocyclic structures in polymers. Moreover, the peaks of the suspected ring structure (M) are more scattered in Figure 1a than in Figure 1b. The signals from −37.0 to −38.5 ppm are still significant and distinct, demonstrating that the macromolecular bridges structure of product D01 catalyzed by a high concentration of catalyst is more complex and more numerous. However, the analogous ring structure peak of product D04 was concentrated at −35.5 ppm and the signal was weaker after −38.0 ppm, revealing that a relatively small number of macromolecular bridges were produced in the polymers synthesized with a low catalyst concentration.

Compared to the distinct signal of the analogous ring structure of product D01, the signal of the analogous ring structure of product D08 (Figure 1c) showed multiple peaks between −35 and −38.5 ppm, indicating that the macromolecular bridges of the prepared polymer at a higher temperature were disordered and uneven. The analogous ring structure signal of product D11 was evenly distributed near −35.5 ppm compared with that of product D04, but was approximately the same in the ring structure signal region of −37 to −38.5 ppm. This shows that the effect on the macromolecular bridges is insignificant between 20% and 35% of reactant concentration. The T_2_ units [R‘‘OSi(OSi)_2_R] are divided into two broad signals (d_1,2_), which distinguish the structure of the straight chain from that of the molecular bridge, and provide evidence for intramolecular bonding between the linear chains and molecular bridges. 

**Figure 2:**
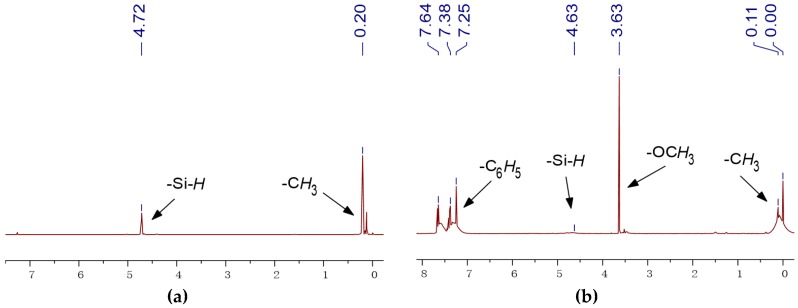
^1^H NMR spectra of HCS (**a**) and D01 (**b**).

The ^1^H NMR spectra (see Figure 2) show that the active hydrogen on HCS was consumed in large amounts. Furthermore, the presence of phenyl and significant attenuation of the Si‒H bond signal revealed that dehydrocarbon polycondensation proceeded successfully. Thus, it is deduced that the consumption of Si‒H bonds by polycondensation reaction is consistent with our expectation, subsequently leading to the introduction of phenyl groups. The NMR signal of the alkoxy groups indicated that the polymer received a number of functional groups owing to the exchange reaction between the reactants, which lays the foundation for its other reactions as a prepolymer.

**Figure 3:**
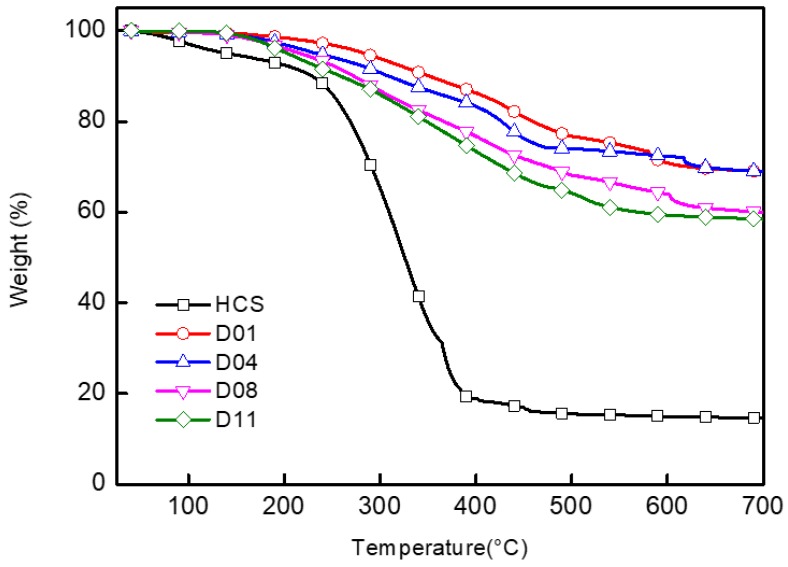
TGA of HCS (*T*_5%_ = 141.5 °C, *T*_10%_ = 229.3 °C, *R*_w_ = 14.63%), D01 (*T*_5%_ = 284.7 °C, *T*_10%_ = 351.8 °C, *R*_w_ = 68.94%), D04 (*T*_5%_ = 235.2 °C, *T*_10%_ = 309.5 °C, *R*_w_ = 69.0%), D08 (*T*_5%_ = 217.5 °C, *T*_10%_ = 272.8 °C, *R*_w_ = 59.96%), D11 (*T*_5%_ = 203.7 °C, *T*_10%_ = 259.0 °C, *R*_w_ = 58.45%).

The refractive index and thermal properties of the polymers were studied, and the results are presented in Table 3 and Figure 3. It is well known that the introduction of phenyl groups would enhance the refractive index of pure polysiloxanes [32]. The refractive indexes of the obtained poly(phenyl-substituted siloxanes/silsesquioxanes) ranged from 1.5056 to 1.5191, indicating their potential application as optical materials. The presence of molecular bridges like D_2_^Ph2^ structures and Si–O–Si chain units in poly(phenyl-substituted siloxanes/silsesquioxanes) is also supported by its heat resistance performance. Since the main chain consists of pure Si–O bonds, polydsiloxane is susceptible to unbuttoned and thermal rearrangement degradation at high temperatures [33,34] whereas the phenyl-substituted polymers with complex structures have excellent thermal stability. Compared with HCS, the temperature at 5% mass loss (*T*_5%_) of B05 was higher by 182.5 °C, and the *R*_w_ (residual weight ratio) increased by 5.17 times from 14.63% to 75.60%. This shows that the high-molecular weight polymers obtained at a high catalyst concentration and low temperature have more phenyl substituents and a greater degree of cross-linking. The thermal stability of D08 obtained at a high temperature (60 °C) is worse than that of D01, which reveals that significant difference is caused by a higher degree of cross-linking. A low molecular weight implies a low degree of crosslinking. The thermal properties as well as the molecular weights of the polymers at low reagent concentrations are not significantly different, which indicates that their structures are not very different. Under the same reaction conditions, the thermal stability of the reaction products D01‒D08 and D11 of DPDMS are lower than that of the reaction products B01‒B08 and B11 of DPDES. The GPC data show that the molecular weights of the D-series products are generally larger than that of the B-series products; however, the molecular weight distribution of the D series is generally wider than that of the B series. Thus, the lower thermal decomposition temperature may be because of the small molecules.

## 4. Conclusions

Dehydrocarbon polycondensation between diphenyldialkoxysilanes and HCS catalyzed by B(C_6_F_5_)_3_ can yield the expected poly(phenyl-substituted siloxanes/silsesquioxanes). The ideal conditions for obtaining stable polymers with complex structures are low temperature (0 °C) to room temperature (25 °C) at which the polycondensation reaction can be promoted, high catalyst concentration (0.8 mmol·L^−1^) at which the product can attain a high molecular weight, and appropriate reactant concentration (35%) at which the reaction can avoid gelling and excessive consumption of toluene. The aforementioned reaction condition implies that the preparation method is both energy-efficient and environmentally friendly, and avoids by-products that can severely erode the instruments. The presence of side groups or molecular bridges in the poly(phenyl-substituted siloxanes/silsesquioxanes) significantly improved the refractive index and thermal stability of the polymers. The poly(phenyl-substituted siloxane/silsesquioxane), which retains Si‒H bonds and alkoxy groups, can be reapplied to addition- and condensation-type curing. We are not content to develop only this type of poly(phenyl-substituted siloxane/silsesquioxane) but aim to develop another type of poly(phenyl-substituted siloxane/silsesquioxane) and investigate its properties after curing.

## 5. Patents

The research results have been patented and the application number is CN201710611199.5.

## Figures and Tables

**Table 1:** Specific reaction conditions and gel permeation chromatography (GPC) data of polycondensation of diphenyldialkoxysilane with hydrogen-containing siloxanes (HCS) in toluene.

No.	Temperature (°C)	Catalyst Concentration (mmol·L^−1^)	Reactant Concentration (wt %)	Yield (%)	*M*_w_ (KDa)	*M*_w_/*M*_n_
B01	25	0.8	35	71.42	26.13	3.57
B02	25	0.4	35	79.00	11.64	2.11
B03	25	0.2	35	79.48	7.39	1.68
B04	25	0.1	35	86.48	6.10	1.52
B05	0	0.8	35	72.32	32.17	3.83
B06	10	0.8	35	78.30	65.09	7.08
B07	40	0.8	35	76.74	20.63	3.17
B08	60	0.8	35	73.57	14.38	2.50
B09	25	0.1	65	gel	/	/
B10	25	0.1	50	gel	/	/
B11	25	0.1	20	80.60	8.78	2.01
D01	25	0.8	35	60.60	40.52	4.76
D02	25	0.4	35	62.74	9.10	3.73
D03	25	0.2	35	74.24	7.25	3.88
D04	25	0.1	35	78.68	4.77	3.85
D05	0	0.8	35	72.19	48.03	4.50
D06	10	0.8	35	63.29	75.60	5.37
D07	40	0.8	35	73.47	34.36	3.28
D08	60	0.8	35	73.57	22.52	4.72
D09	25	0.1	65	gel	/	/
D10	25	0.1	50	gel	/	/
D11	25	0.1	20	83.82	5.77	1.53

B01~B11 represent a series of experiments involving Piers–Rubinsztajn (PR) reaction between diphenyldiethoxysilane (DPDES) and HCS in the above-mentioned specific experimental conditions. D01~D11 represent a series of experiments involving PR reaction between diphenyldimethoxysilane (DPDMS) and HCS in the above-mentioned specific experimental conditions. The reactant concentration is equal to the sum of the masses of diphenyldialkoxysilane and HCS divided by the sum of the masses of diphenyldialkoxysilane, HCS, and toluene. For example, B01: 17 g HCS, 29.64 g DPDES, 86.62 g toluene.

**Table 2:** Key parameters of various reactants and catalyst optimized at B3LYP/6-311G or STO-3G level.

Species	Bond Length (Å)	Atomic Charge	Species	Bond Length (Å)	Atomic Charge
DPDMS	R_O(24)-C(26)_ = 1.4249	q_Si(1)_ = 0.7899	DPDES	R_O(24)-C(29)_ = 1.4306	q_Si(1)_ = 1.1188
	R_O(25)-C(30)_ = 1.4249	q_O(24)_ = −0.4336		R_O(25)-C(26)_ = 1.4307	q_O(24)_ = −0.3791
	R_O(24)-Si(1)_ = 1.666	q_O(25)_ = −0.4335		R_O(24)-Si(1)_ = 1.6628	q_O(25)_ = −0.3789
	R_O(25)-Si(1)_ = 1.666	q_C(26)_ = 0.2844		R_O(25)-Si(1)_ = 1.6628	q_C(26)_ = 0.2034
		q_C(30)_ = 0.2844			q_C(29)_ = 0.2035
miniHCS /B(C_6_F_5_)_3_	R_H(37)-Si(36)_ = 1.4474	q_B_ = 0.2745	miniHCS	R_H(3)-Si(2)_ = 1.4523	q_Si(2)_ = 0.7965
	R_H(38)-Si(36)_ = 1.4546	q_Si(36)_ = 0.5359		R_H(4)-Si(2)_ = 1.453	q_Si(5)_ = 0.7940
	R_H(40)-Si(39)_ = 1.4476	q_Si(39)_ = 0.5743		R_H(6)-Si(5)_ = 1.4531	q_H(3)_ = −0.1519
	R_H(41)-Si(39)_ = 1.4492	q_H(37)_ = −0.1314		R_H(7)-Si(5)_ = 1.4527	q_H(4)_ = −0.1499
		q_H(38)_ = −0.1502			q_H(6)_ = −0.1447
		q_H(40)_ = −0.1153			q_H(7)_ = −0.1507
		q_H(41)_ = −0.1149	B(C_6_F_5_)_3_		q_B_ = 0.802

((CH_3_)_3_SiOSiH_2_O)_2_ (miniHCS) was used as a simplified model for HCS to simplify the calculation. MiniHCS/B(C_6_F_5_)_3_ and miniHCS optimized at B3LYP/STO-3G level. DPDMS, DPDES, and B(C_6_F_5_)_3_ optimized at B3LYP/6-311G level. Atomic charges with hydrogens of DPDMS and DPDES summed into heavy atoms of C bonded to H. The numbered elements’ (Si, O, C) specific location in DPDMS: (1)Si-(24)O-(26)CH_3_, (1)Si-(25)O-(30)CH_3_. The numbered elements’ (Si, O, C) specific location in DPDES: (1)Si-(24)O-(29)CH_2_CH_3_, (1)Si-(25)O-(26)CH_2_CH_3_. The numbered elements’ (Si, H) specific location in miniHCS/B(C_6_F_5_)_3_: (37)H-(36)Si-(38)H, (40)H-(39)Si-(41)H. The numbered elements’ (Si, H) specific location in miniHCS: (3)H-(2)Si-(4)H, (6)H-(5)Si-(7)H. (see supplementary Image 1–5 about the specific calculation models).

**Table 3:** Refractive index and thermal properties of the products

No.	Refractive Index	*T*_5%_ (°C)	*T*_10%_ (°C)	*R*_W_ (%)
HCS	1.4123	141.5	229.3	14.6
B01	1.5190	309.8	374.5	72.4
B02	1.5189	302	362.2	72.32
B03	1.5186	290.3	342.7	65.43
B04	1.5078	270.8	332.7	68.74
B05	1.5191	324	380.8	75.60
B06	1.5190	316.8	368	65.54
B07	1.5163	263	335.8	70.38
B08	1.5151	233.7	306.5	66.66
B09	—	—	—	—
B10	—	—	—	—
B11	1.5056	265	369.3	71.47
D01	1.5173	284.7	351.8	68.94
D02	1.5160	265.5	319.7	64.66
D03	1.5142	260.7	339.3	72.51
D04	1.5119	235.2	309.5	68.99
D05	1.5162	280.5	371.7	75.92
D06	1.5168	273.2	356.2	72.81
D07	1.5159	250.2	327	73.92
D08	1.5151	217.5	272.8	59.96
D09	—	—	—	—
D10	—	—	—	—
D11	1.5159	203.7	259	58.45

Temperature and *R*_w_ means residual weight ratio *T*_5%_ means 5% thermal decomposition temperature, *T*_10%_ means 10% thermal decomposition temperature and *R*_w_ means residual weight ratio.

## Supplementary Materials

The following are available online at http://www.mdpi.com/2073-4360/10/9/1006/s1, Image S1: The calculation model of DPDMS, Image S2: The calculation model of DPDES, Image S3: The calculation model of B(C_6_F_5_)_3_, Image S4: The calculation model of miniHCS, Image S5: The calculation model of B(C_6_F_5_)_3_ and miniHCS, Table S1: GPC data of the products, Figure S1: Thermogravimetric analysis of HCS, B01, B02, B03 and B04, Figure S2: Thermogravimetric analysis of HCS, B04 and B11, Figure S3: Thermogravimetric analysis of HCS, B05, B06, B01, B07 and B08, Figure S4: Thermogravimetric analysis of HCS, D01, D02, D03 and D04, Figure S5: Thermogravimetric analysis of HCS, D04 and D11, Figure S6: Thermogravimetric analysis of HCS, D05, D06, D01, D07and D08, Figure S7: ^1^H NMR of HCS, Figure S8: ^1^H NMR of B01, Figure S9: ^1^H NMR of D01, Figure S10: ^29^Si NMR of HCS, Figure S11: ^29^Si NMR of DPDMS, Figure S12: ^29^Si NMR of DPDES, Figure S13: ^29^Si NMR of B01, Figure S14: ^29^Si NMR of B02, Figure S15: ^29^Si NMR of B03, Figure S16: ^29^Si NMR of B04, Figure S17: ^29^Si NMR of B05, Figure S18: ^29^Si NMR of B06, Figure S19: ^29^Si NMR of B07, Figure S20: ^29^Si NMR of B08, Figure S21: ^29^Si NMR of B11, Figure S22: ^29^Si NMR of D01, Figure S23: ^29^Si NMR of D02, Figure S24: ^29^Si NMR of D03, Figure S25: ^29^Si NMR of D04, Figure S26: ^29^Si NMR of D05, Figure S27: ^29^Si NMR of D06, Figure S28: ^29^Si NMR of D07, Figure S29: ^29^Si NMR of D08, Figure S30: ^29^Si NMR of D11.
