# Supplementary material for: Fabrication of Reactive Poly(Phenyl-Substituted Siloxanes/Silsesquioxanes) with Si‒H and Alkoxy Functional Groups via the Piers–Rubinsztajn Reaction
Source: Polymers (Basel). 2018 Sep 10;10(9):1006. doi: 10.3390/polym10091006 (PMC6403990; doi:10.3390/polym10091006)
Supplement: Supplementary file 1 [file polymers-10-01006-s001.pdf]

Data article

GPC data, NMR spectra, thermogravimetric analysis and the specific calculation models based on the quantum chemical density functional theory (DFT) .

Authors: Minghao Yi

Affiliations: Zhongkai University of Agriculture and Engineering.

Contact email: MHYi0848@163.com

Abstract

GPC data, NMR spectra and thermogravimetric analysis of products and the specific calculation models of various reactants and catalyst based on the quantum chemical density functional theory can be seen in this data article .

Specifications Table *[please fill in right-hand column of the table below]*

Subject area	*Chemistry*
More specific subject area	*Polymer*
Type of data	*image, figure, table*
How data was acquired	*GPC(Waters 1525/2414 chromatograph, America) , NMR(Bruker AVANCE AV 400 MHz spectrometer, Germany), thermogravimetric analysis(Mettler TGA 2 thermogravimetric analyzer, America)*
Data format	*Raw, analyzed*
Experimental factors	*The pretreatments of samples can be found in the experimental section below.*
Experimental features	*The various measurements can be found in the experimental section below.*
Data source location	*Guangzhou, China*
Data accessibility	*The data is with this article*

Value of the data

GPC data allows researchers to understand the effect of different Polycondensation conditions on the molecular weights of the polymers. ^{1}H NMR and ^{29}Si NMR spectra may be possible for researchers to understand the structure of new substances and use them as a reference. TGA data may allow researchers to understand the high thermal stability of these highly phenyl-substituted polymers where phenyl substituents, complex structures such as intramolecular ring structures and molecular bridges composed of D_2^{Ph2}[$(C_6H_5)_2Si(OSi)_2$] structures also existed in. The specific calculation models of various reactants and catalyst based on the quantum chemical density functional theory may help researchers understand the optimal configuration and the number of specific elements of the compound in the research article.

Data

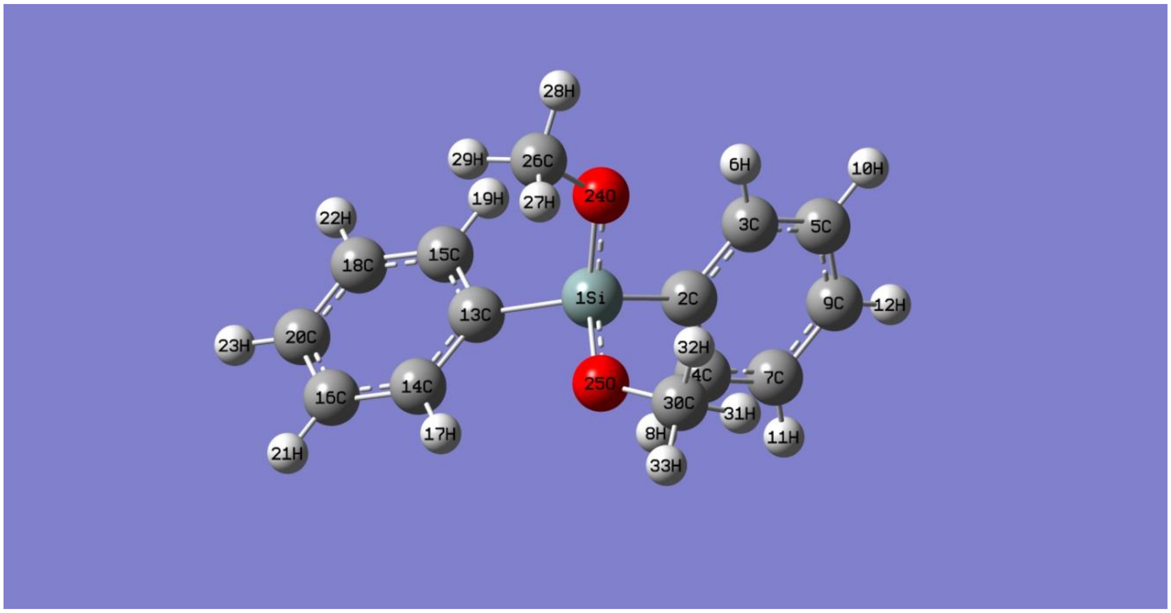

Image S1. *The calculation model of DPDMS*

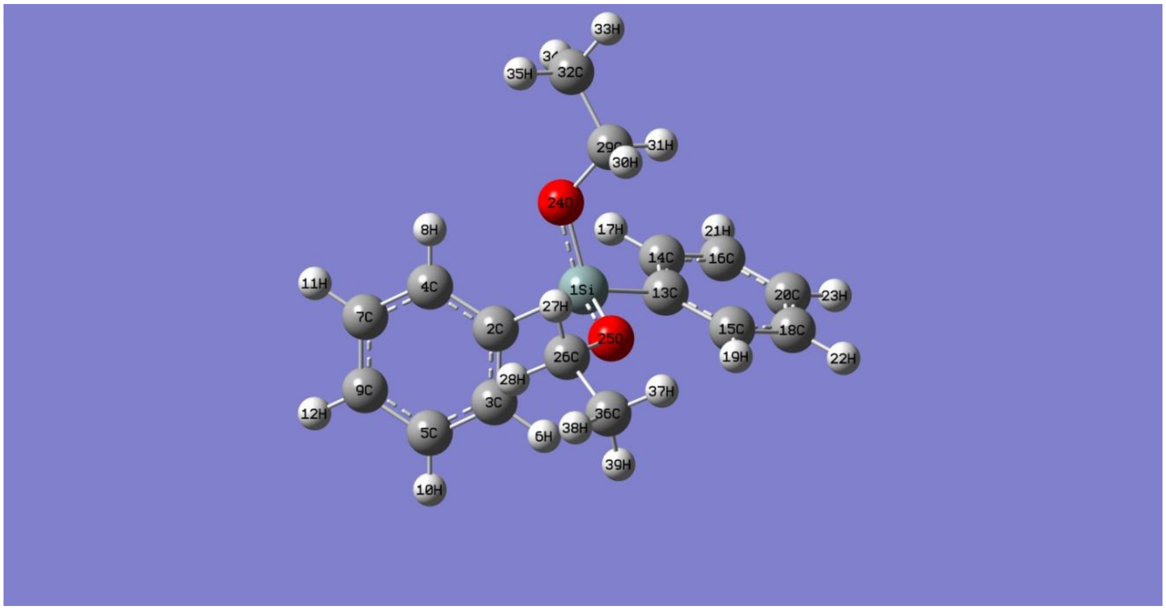

Image S2. *The calculation model of DPDES*

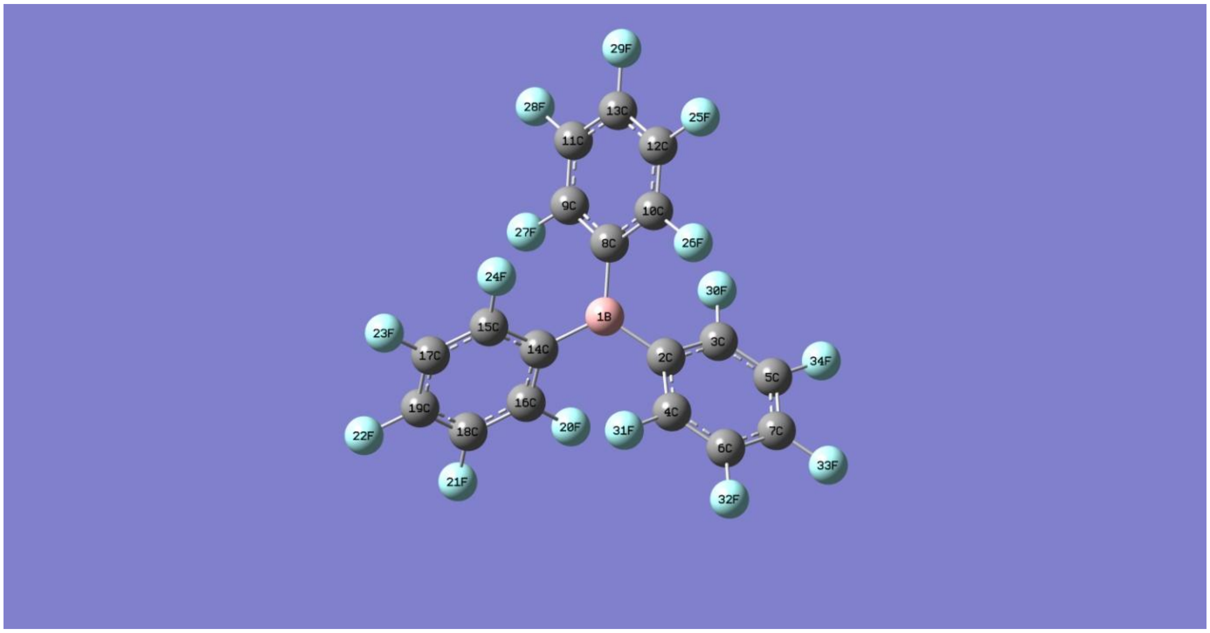

Image S3. *The calculation model of* $B(C_6F_5)_3$

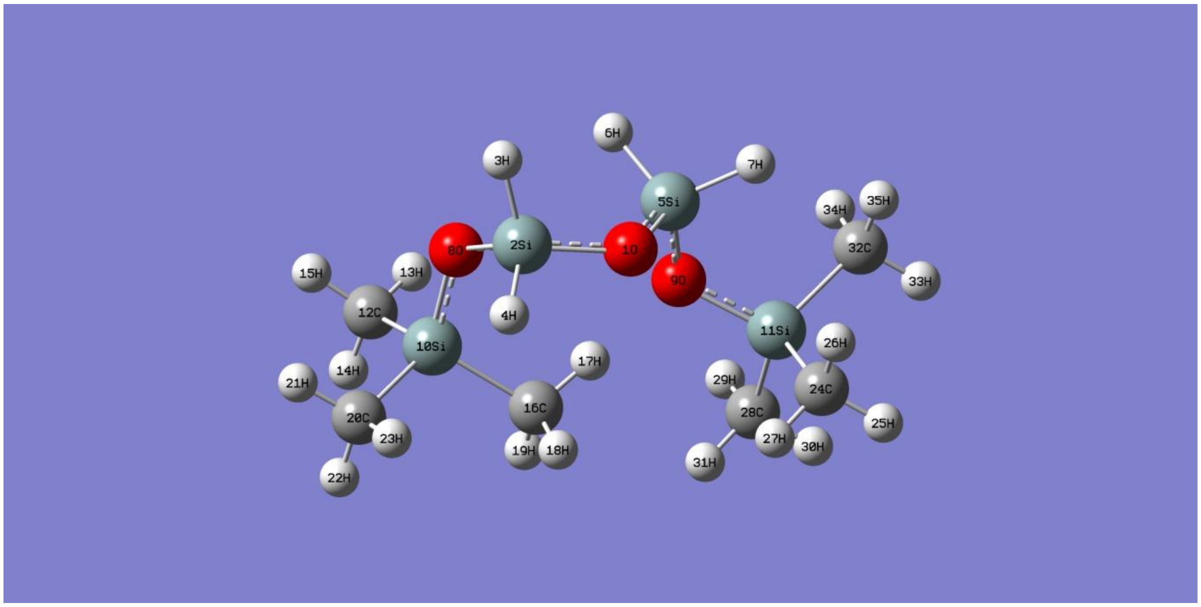

Image S4. *The calculation model of miniHCS*

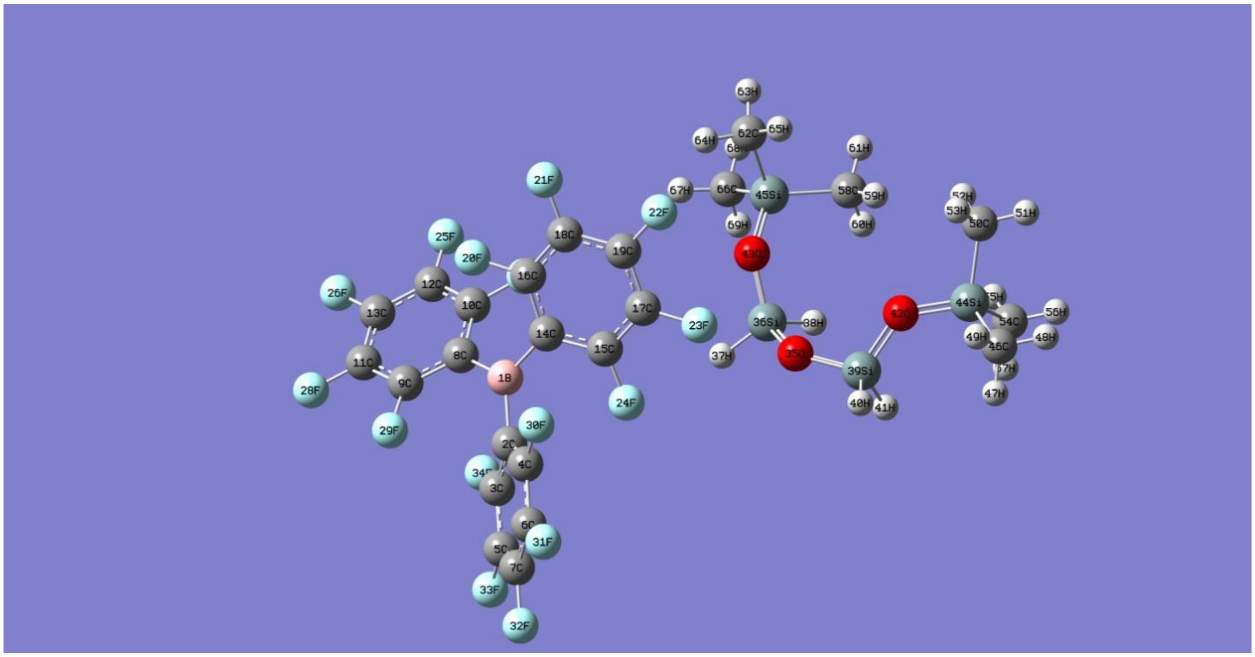

Image S5. *The calculation model of $B(C_6F_5)_3$ and miniHCS*

Table S1. *GPC data of the products*

Sample	Mn (Daltons)	Mw (Daltons)	MP (Daltons)	Mz (Daltons)	Mz+1 (Daltons)	Polydispersity	Mz/Mw	Mz+1/Mw
B01	7328	26128	20313	83743	156308	3.57	3.21	5.98
B02	5521	11644	5836	27381	53341	2.11	2.35	4.58
B03	4393	7389	5035	12012	18041	1.68	1.63	2.44
B04	4001	6100	4561	8916	12187	1.52	1.46	2.00
B05	8041	32170	18966	124391	280199	3.83	3.87	8.71
B06	9190	65086	79049	385706	939751	7.08	5.93	14.44
B07	6516	20628	17336	63506	120442	3.17	3.08	5.84
B08	5751	14376	13981	37230	74498	2.50	2.59	5.18
B11	4375	8778	3788	18815	35684	2.01	2.14	4.07
D01	8518	40519	62295	157570	320375	4.76	3.89	7.91
D02	2439	9099	6371	20327	42461	3.73	2.24	4.67
D03	1866	7253	4478	13882	21960	3.89	1.91	3.03
D04	1283	4773	4195	7678	9865	3.85	1.61	2.07
D05	10682	48025	23691	209095	468042	4.50	4.35	9.75
D06	14070	75596	85360	377795	908866	5.37	5.00	12.02
D07	7439	24364	17336	79748	156875	3.28	3.27	6.44
D08	4766	22516	17510	70331	140789	4.72	3.12	6.25
D11	3772	5766	3528	8408	11259	1.53	1.46	1.95

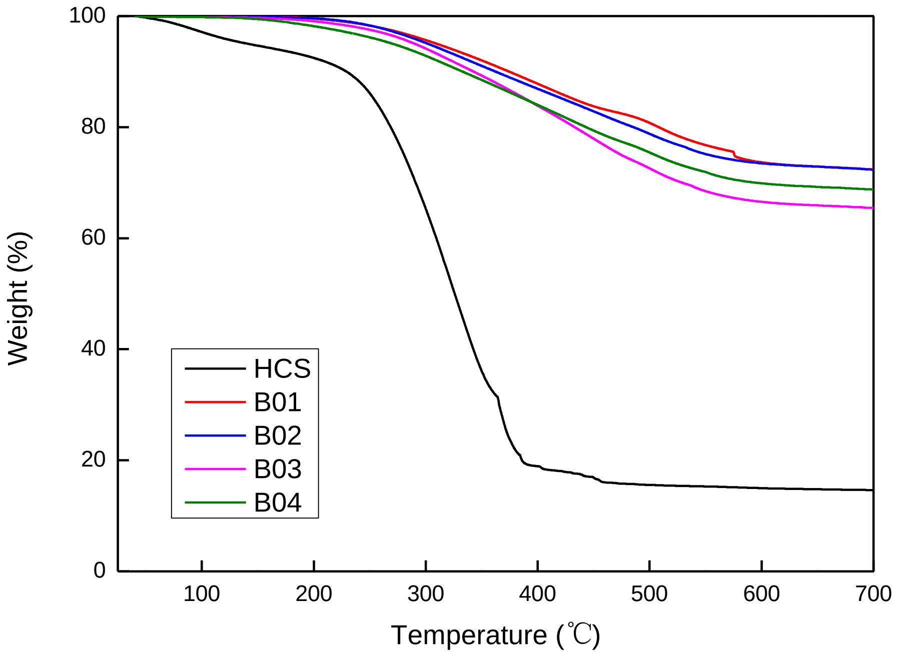

Figure S1. *Thermogravimetric analysis of HCS, B01, B02, B03 and B04.*

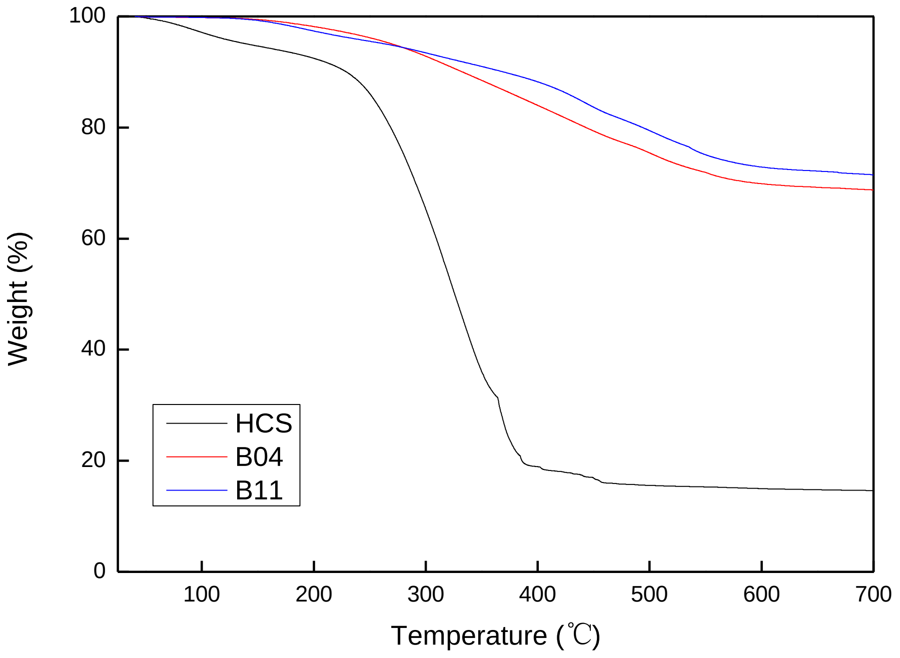

Figure S2. *Thermogravimetric analysis of HCS, B04 and B11.*

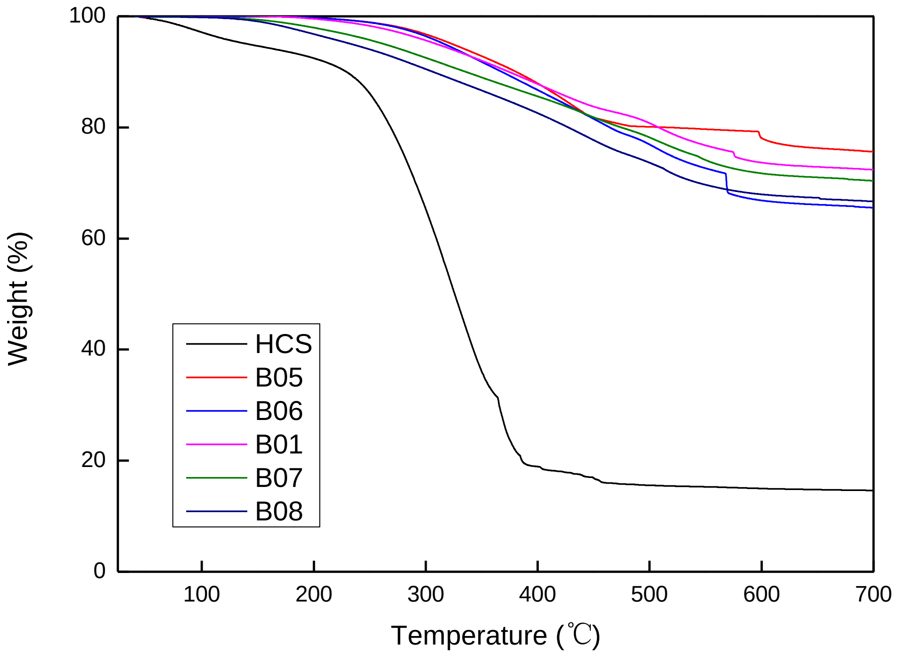

Figure S3. *Thermogravimetric analysis of HCS, B05, B06, B01, B07and B08.*

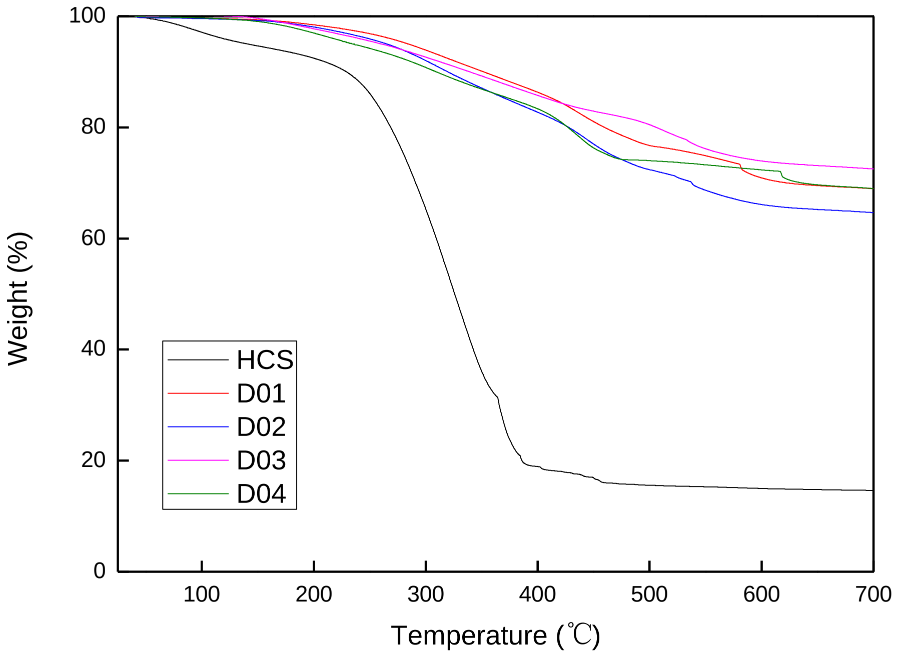

Figure S4. *Thermogravimetric analysis of HCS, D01, D02, D03 and D04.*

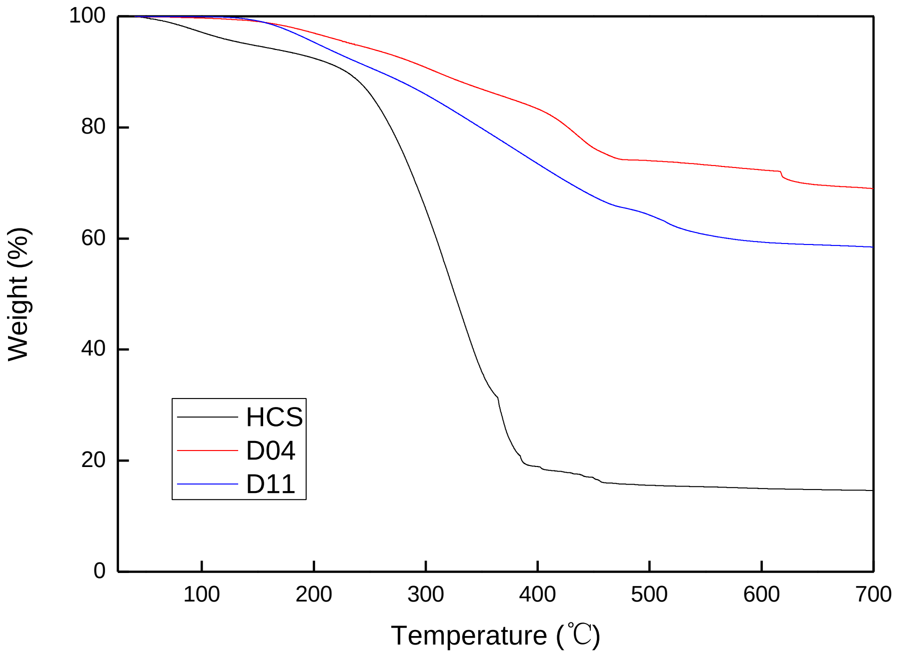

Figure S5. *Thermogravimetric analysis of HCS, D04 and D11.*

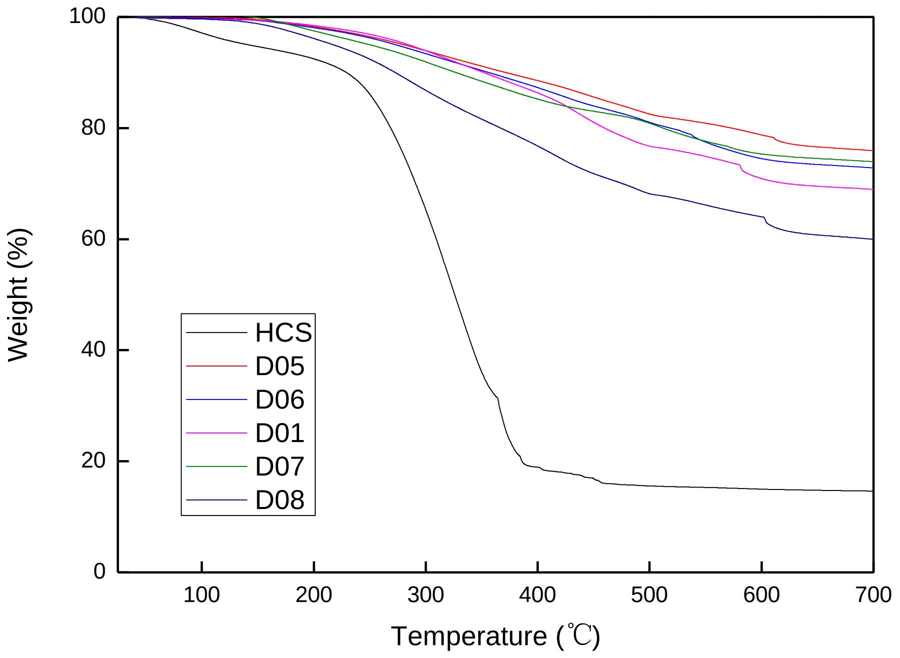

Figure S6. *Thermogravimetric analysis of HCS, D05, D06, D01, D07and D08.*

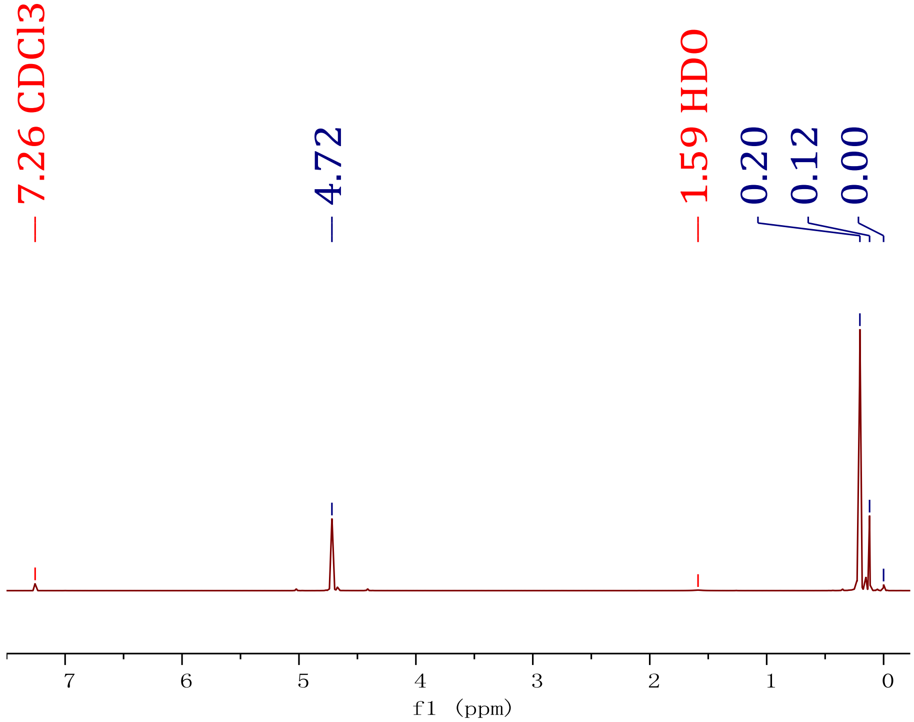

Figure S7. *^{1}H NMR of HCS*

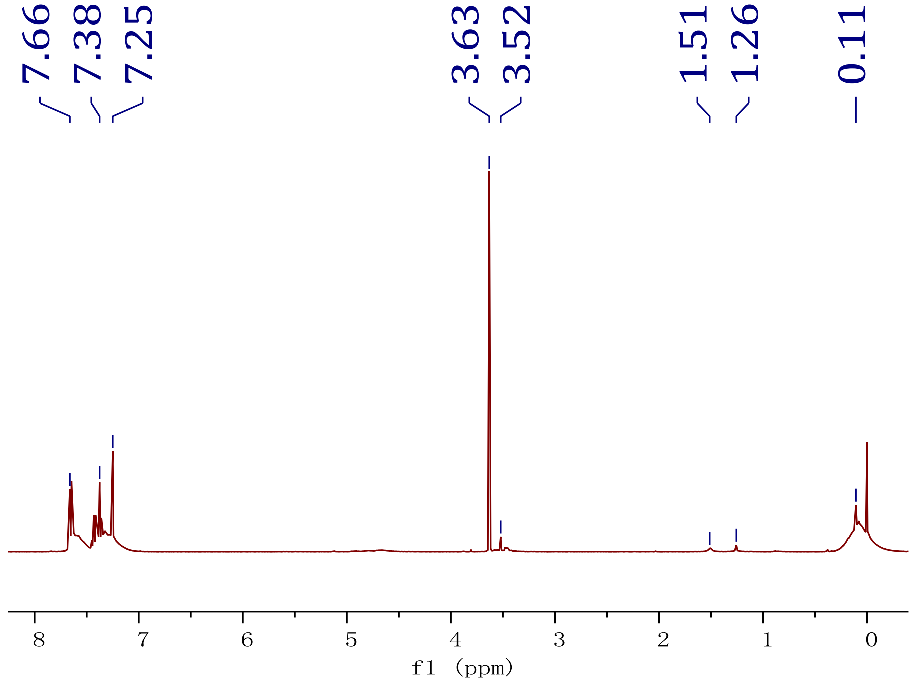

Figure S8. *^{1}H NMR of B01*

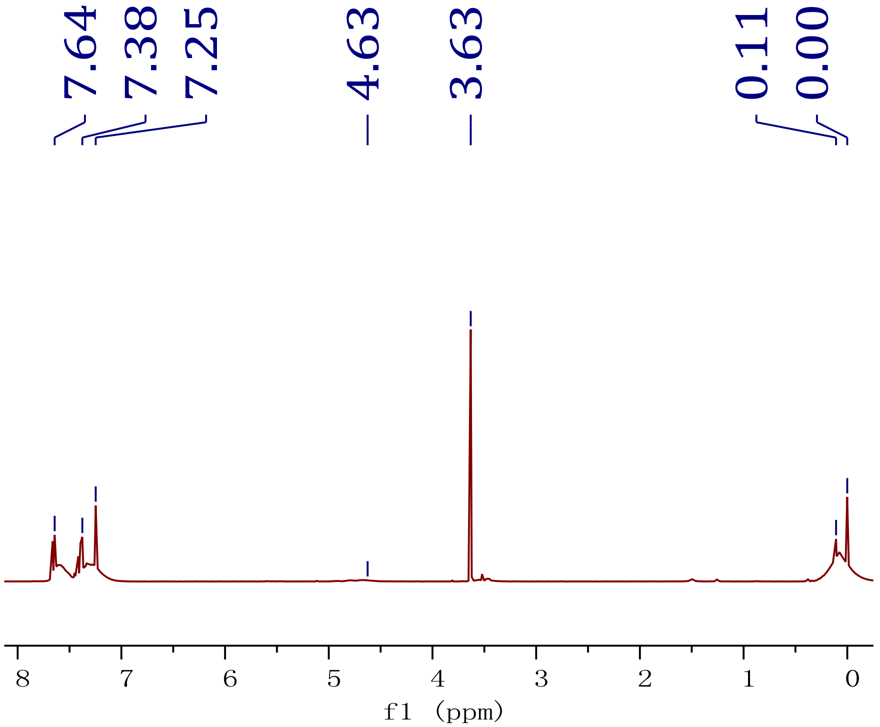

Figure S9. *^{1}H NMR of D01*

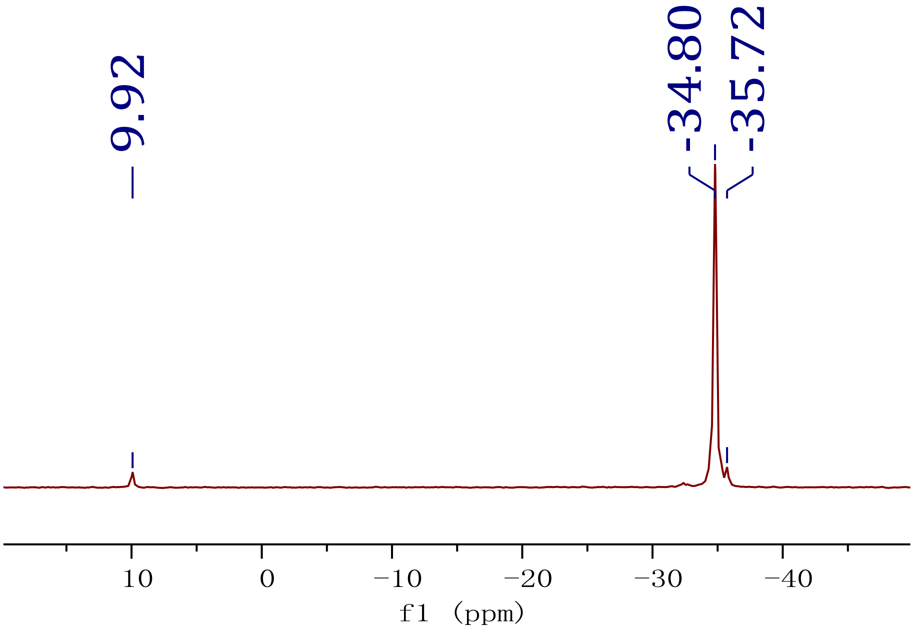

Figure S10. *^{29}Si NMR of HCS*

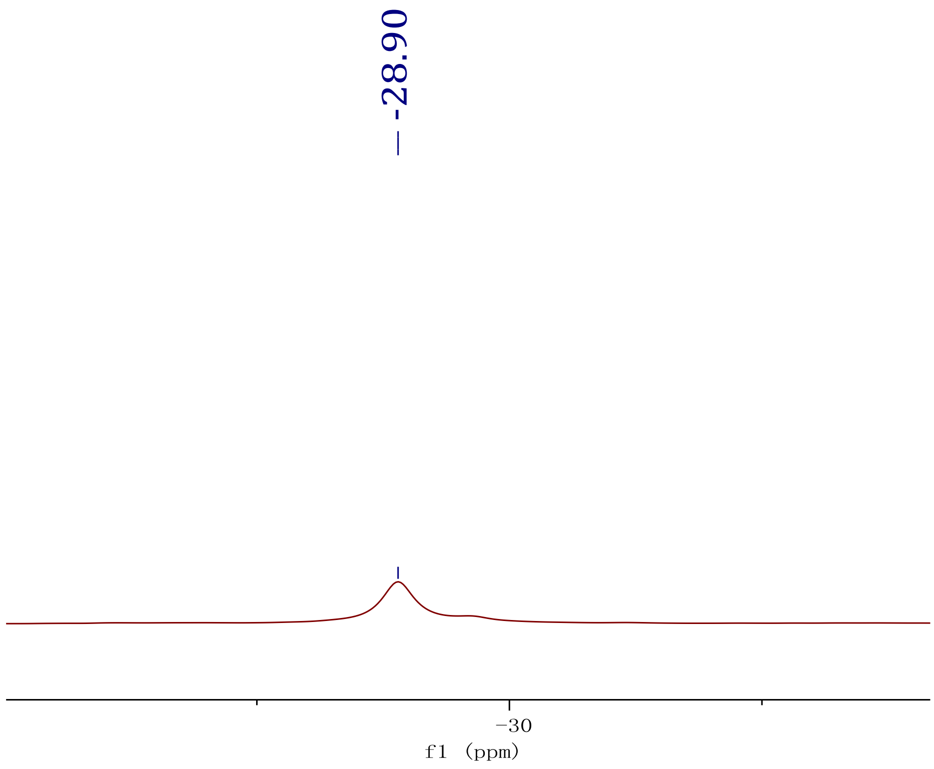

Figure S11. *^{29}Si NMR of DPDMS*

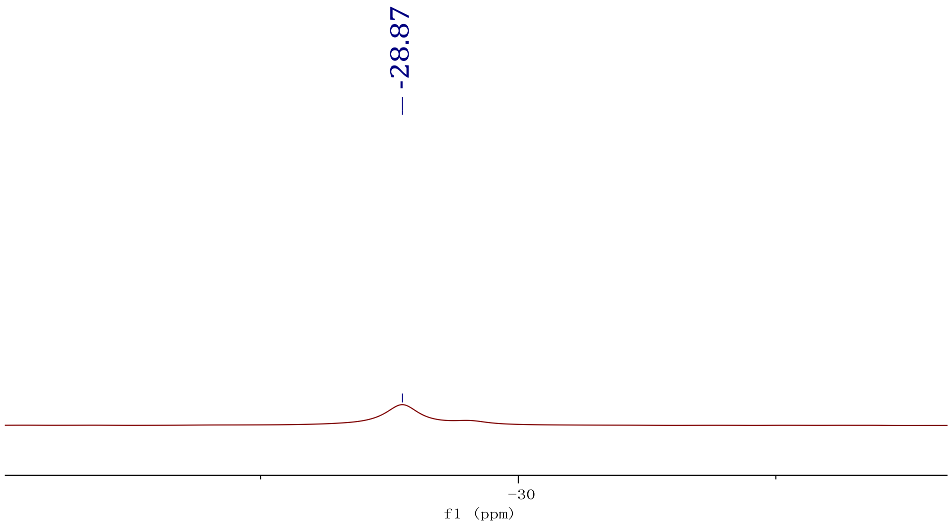

Figure S12. *^{29}Si NMR of DPDES*

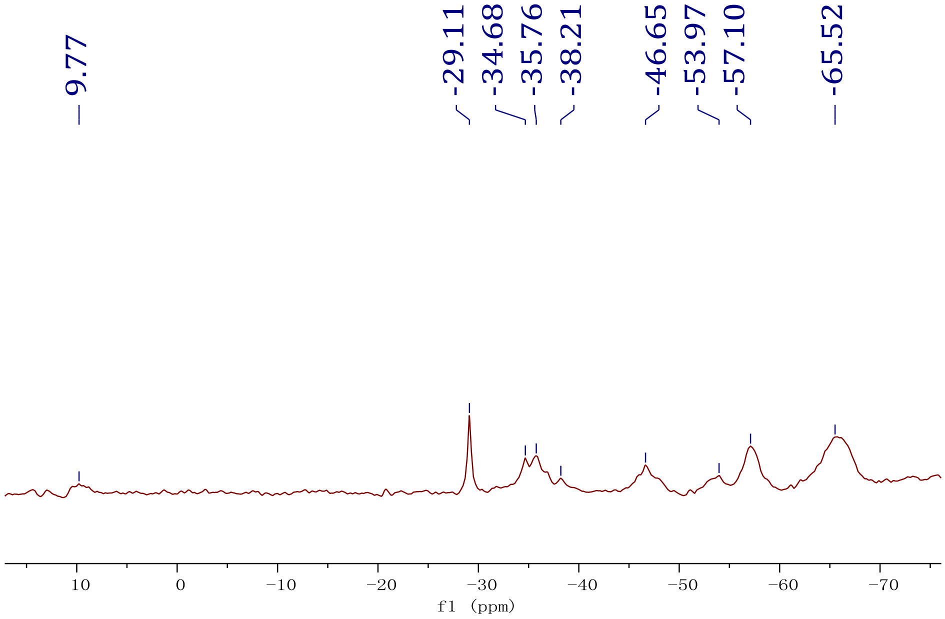

Figure S13. *^{29}Si NMR of B01*

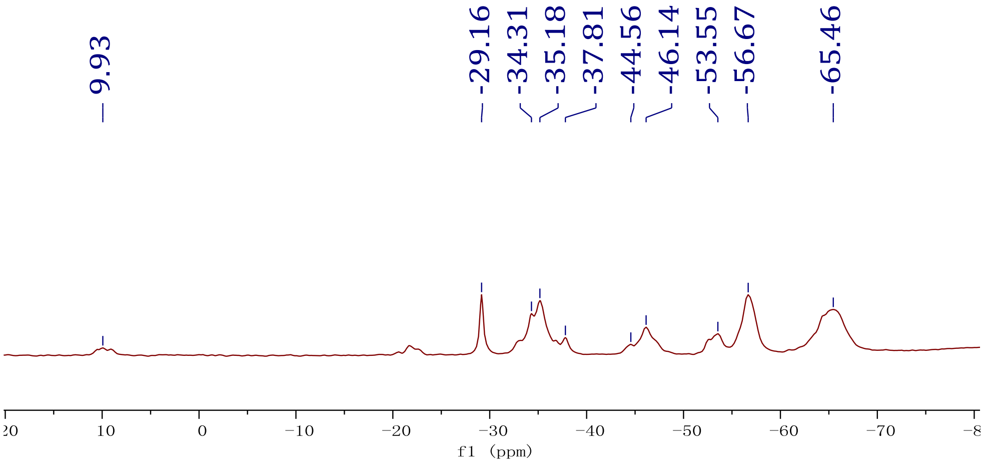

Figure S14. *^{29}Si NMR of B02*

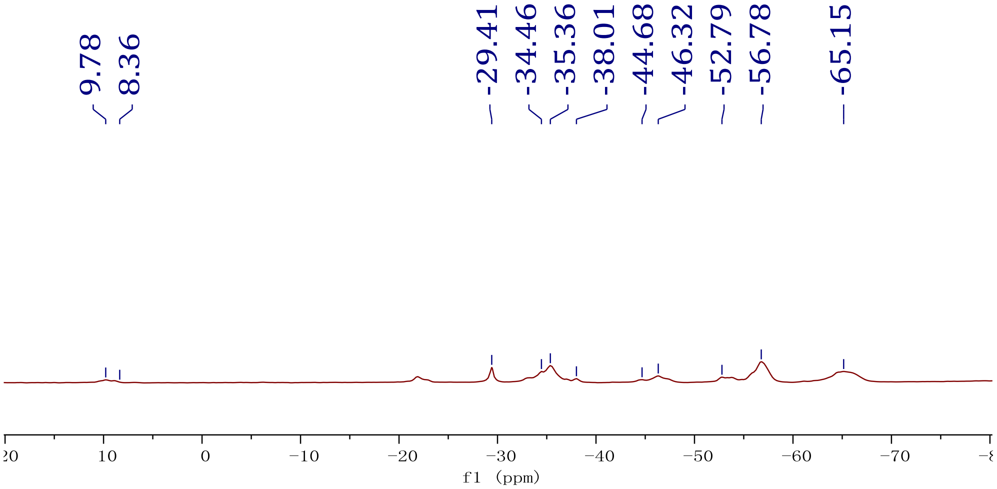

Figure S15. *^{29}Si NMR of B03*

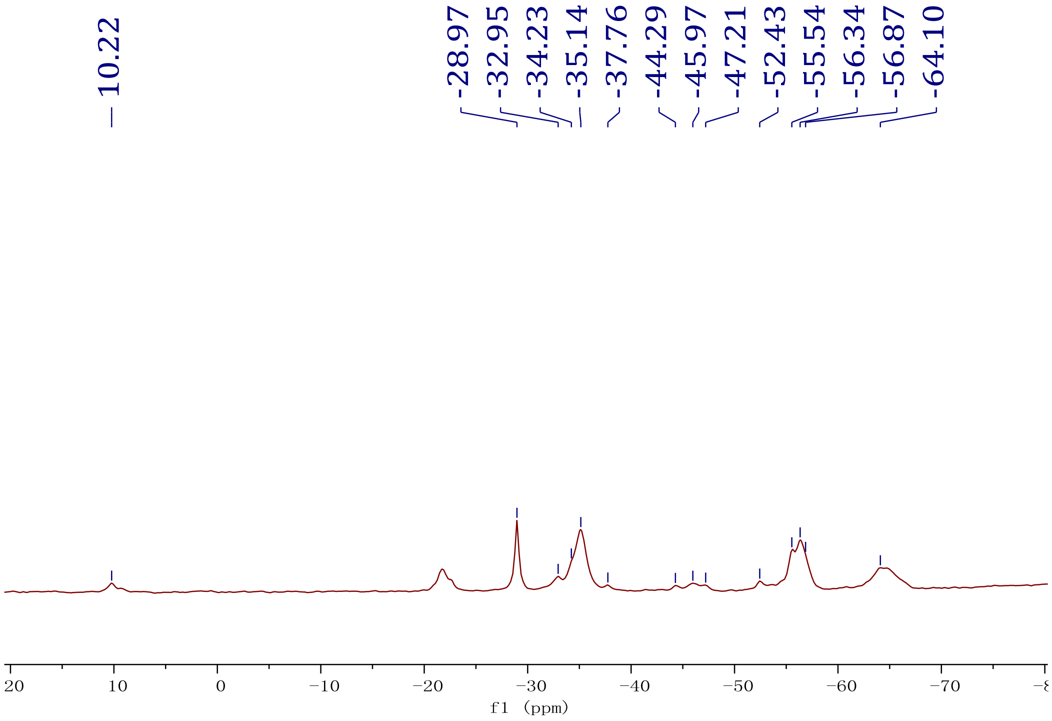

Figure S16. *^{29}Si NMR of B04*

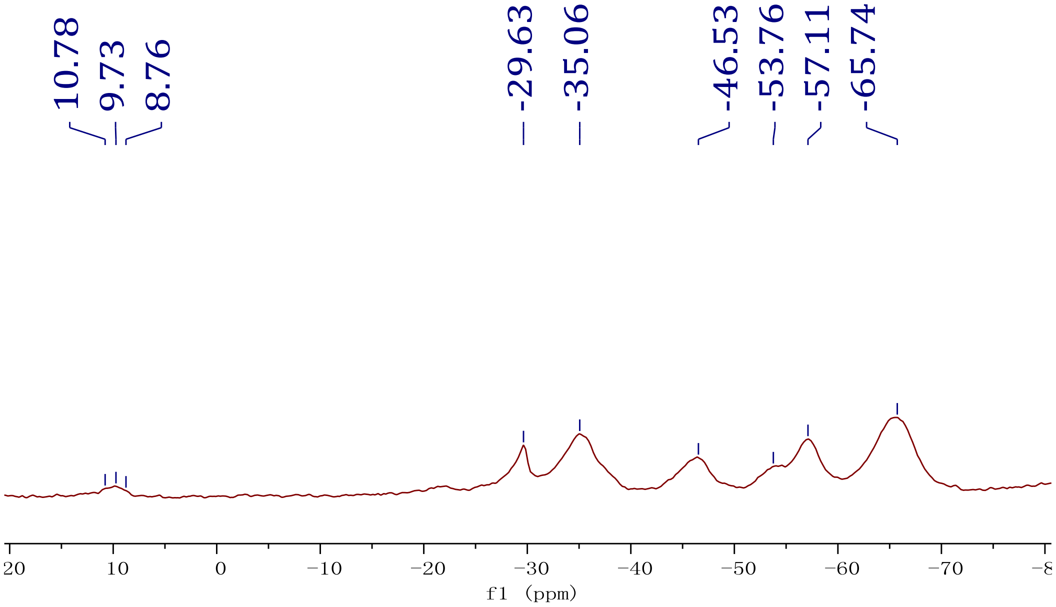

Figure S17. *²⁹Si NMR of B05*

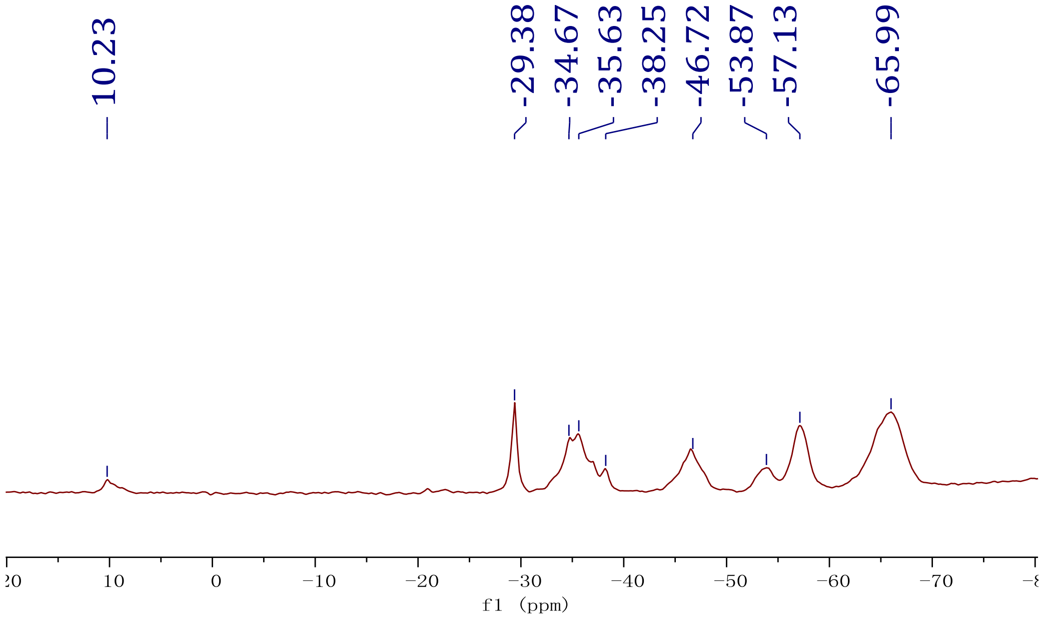

Figure S18. *²⁹Si NMR of B06*

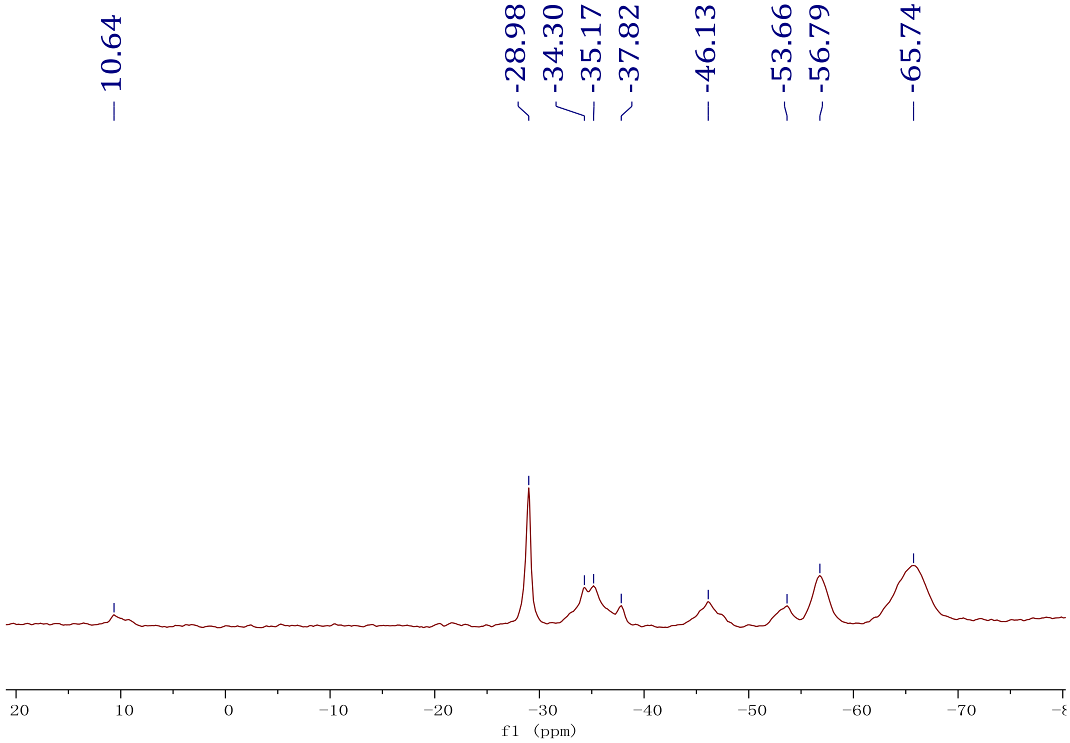

Figure S19. *^{29}Si NMR of B07*

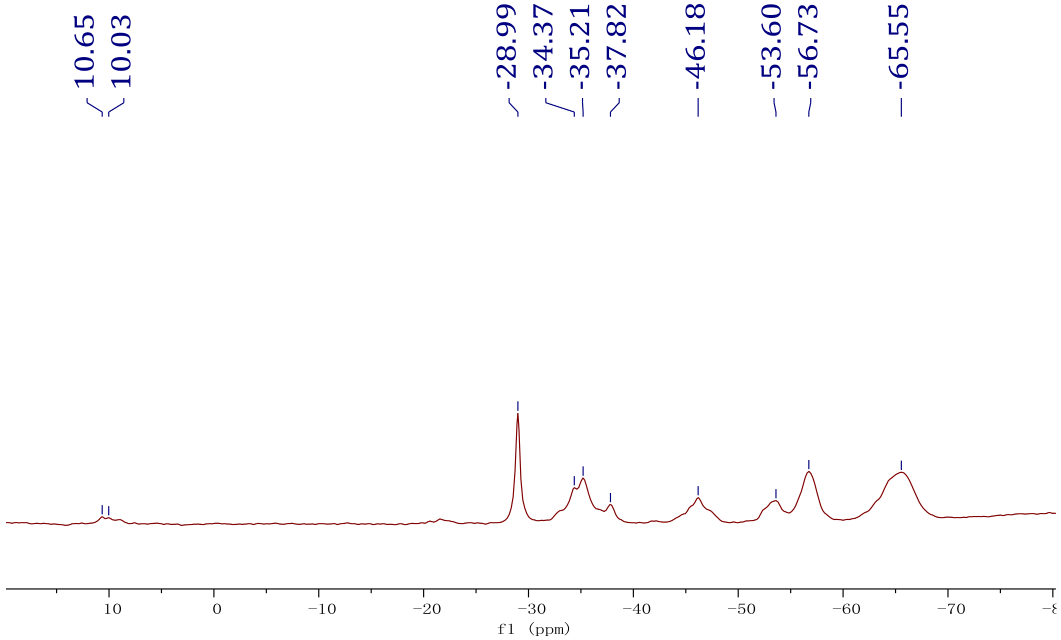

Figure S20. *^{29}Si NMR of B08*

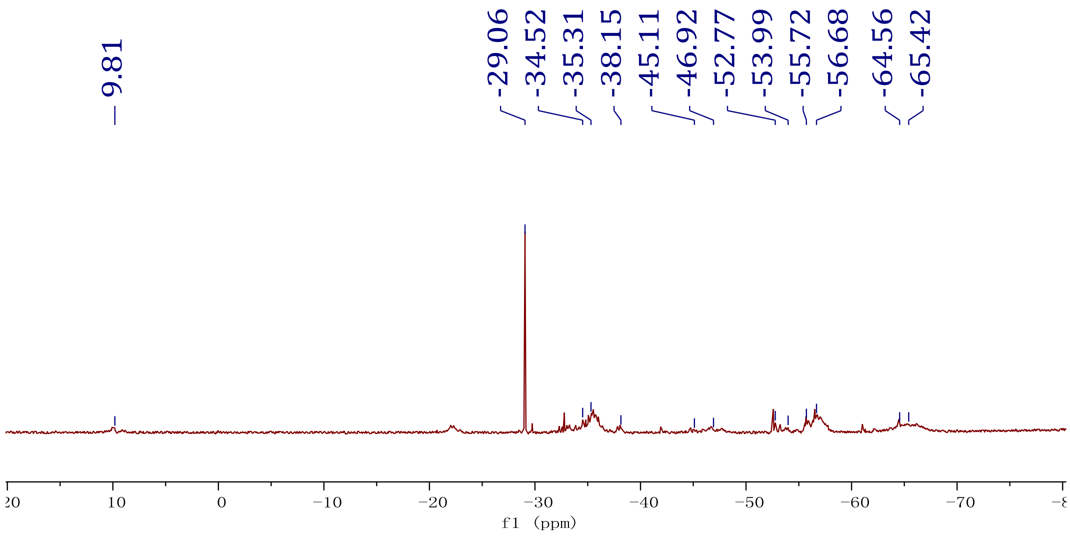

Figure S21. *^{29}Si NMR of B11*

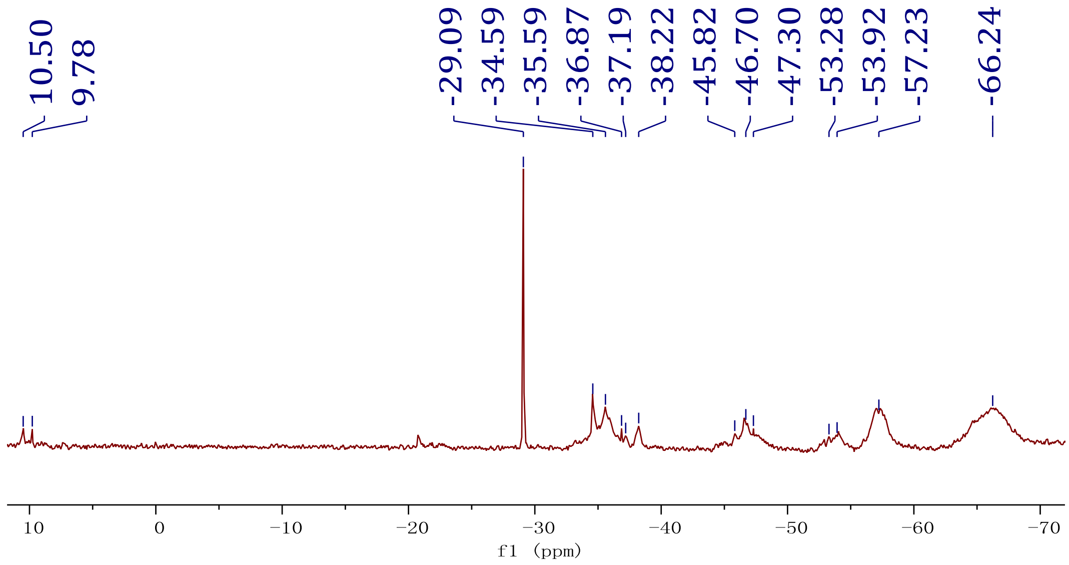

Figure S22. *^{29}Si NMR of D01*

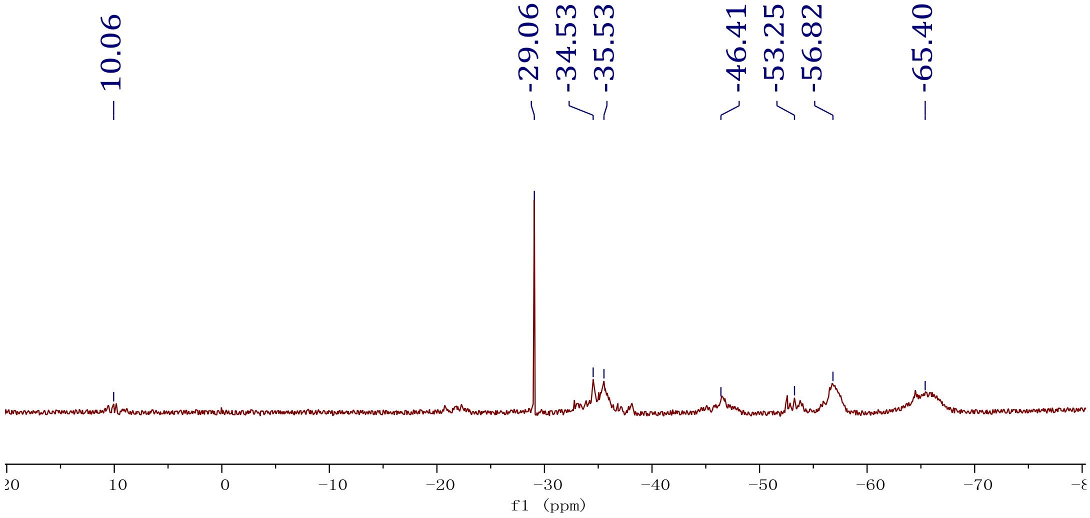

Figure S23. *^{29}Si NMR of D02*

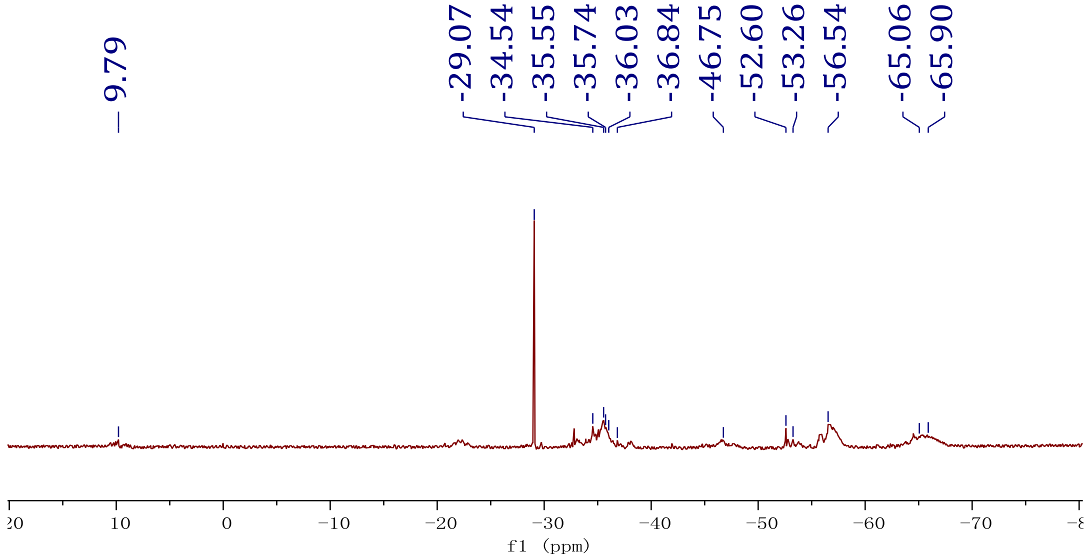

Figure S24. *^{29}Si NMR of D03*

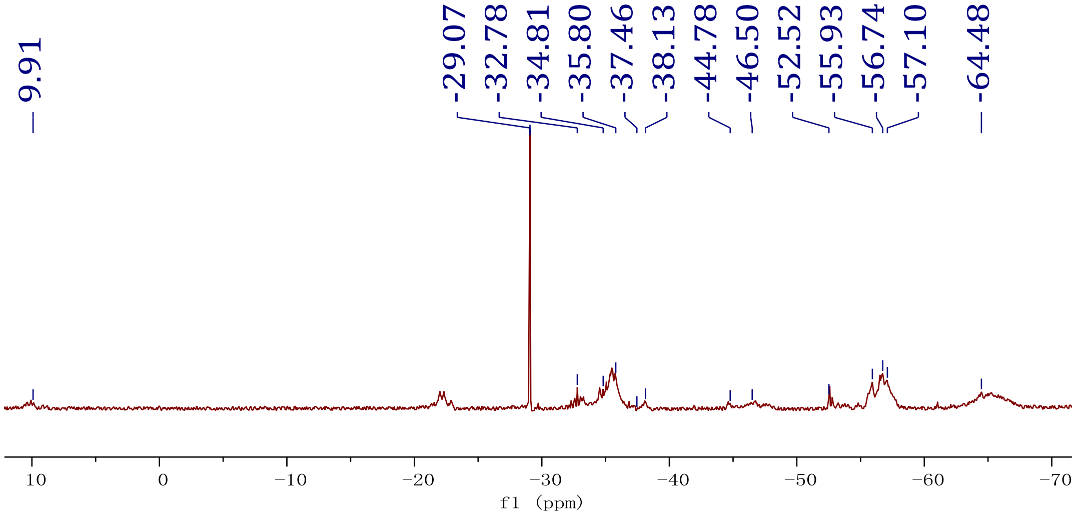

Figure S25. *^{29}Si NMR of D04*

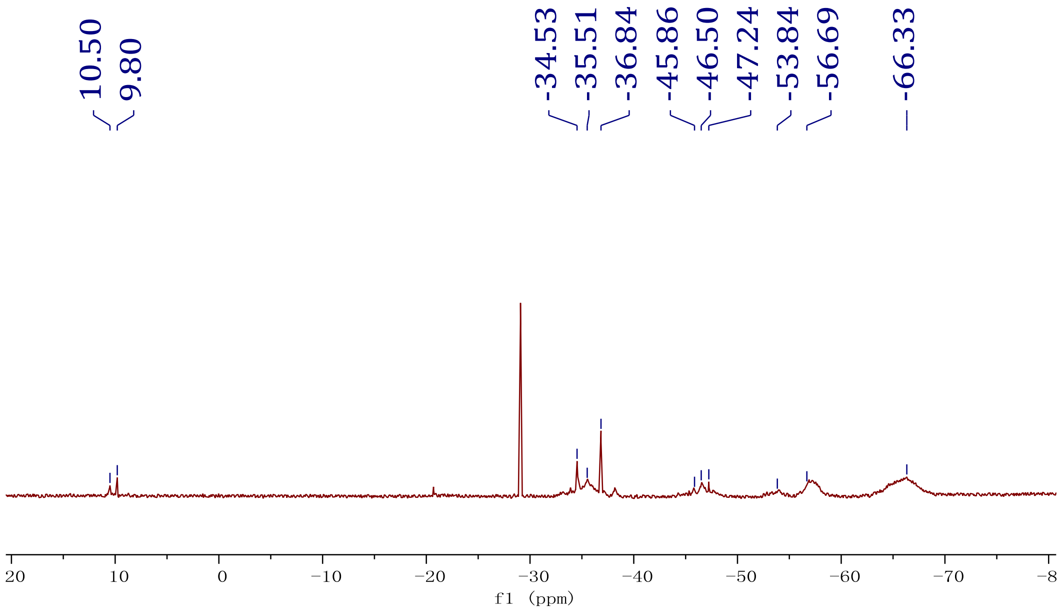

Figure S26. *^{29}Si NMR of D05*

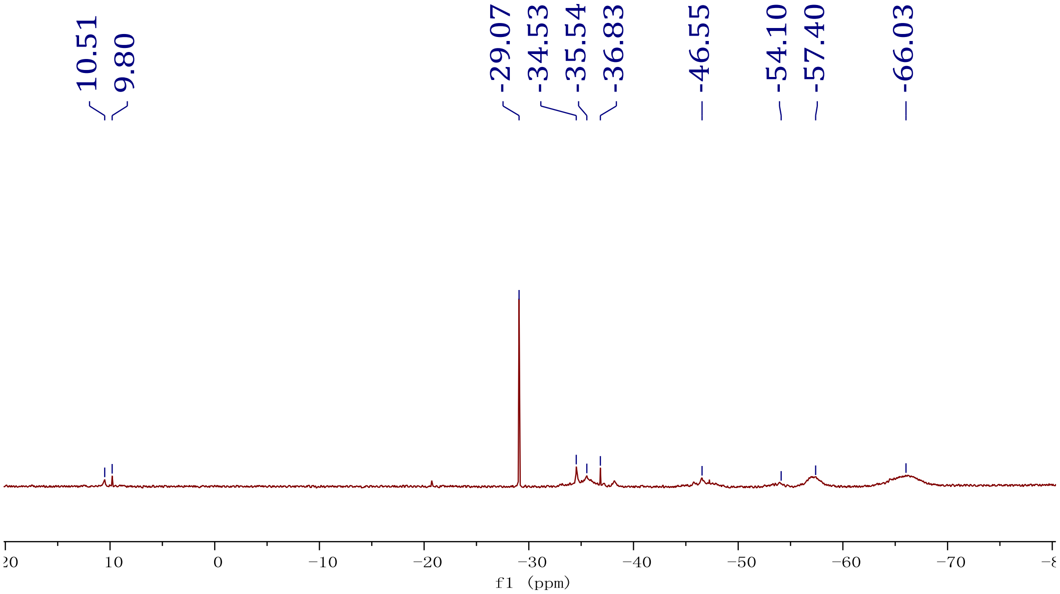

Figure S27. *^{29}Si NMR of D06*

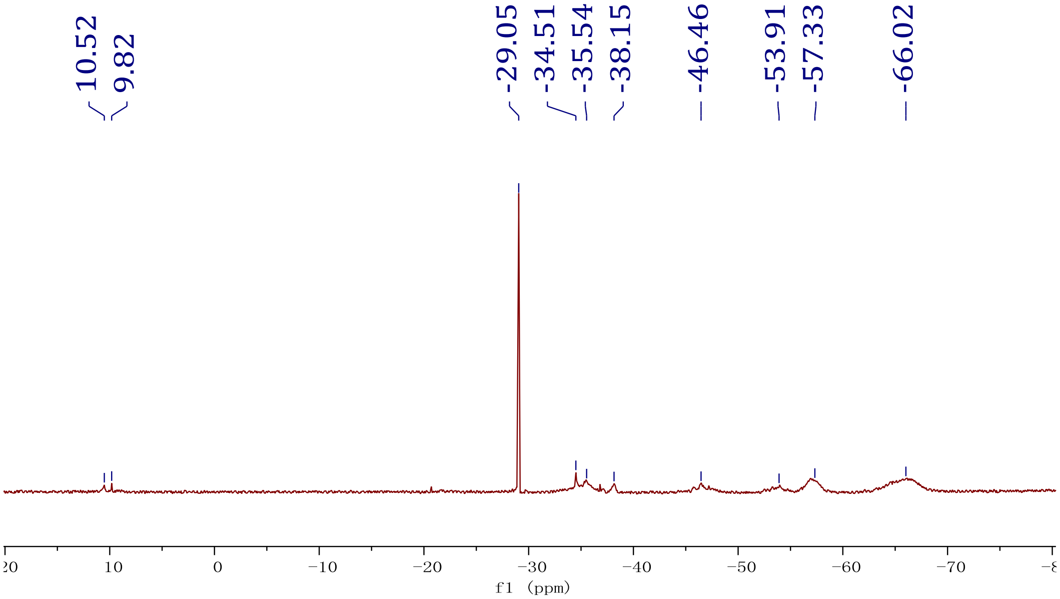

Figure S28. ^{29}Si NMR of D07

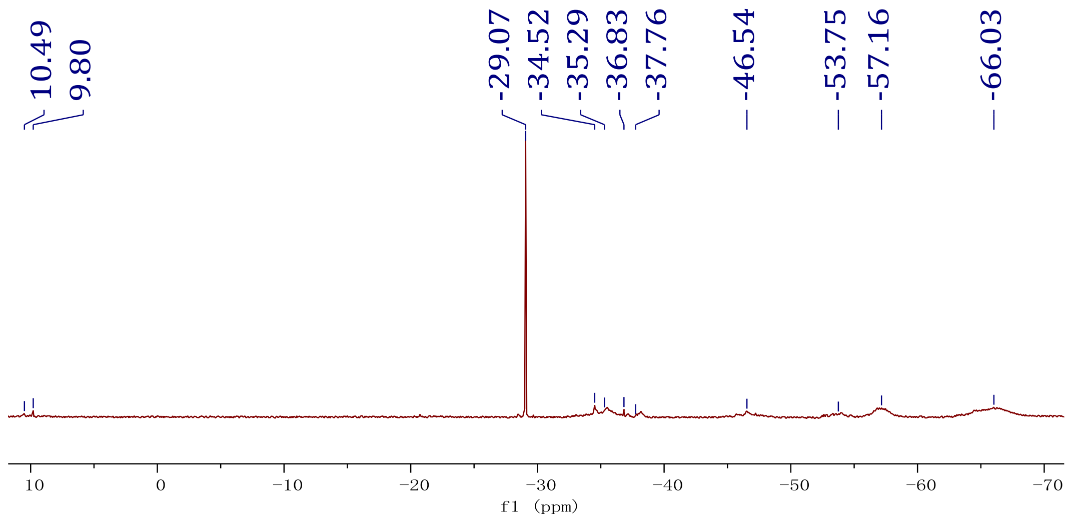

Figure S29. *^{29}Si NMR of D08*

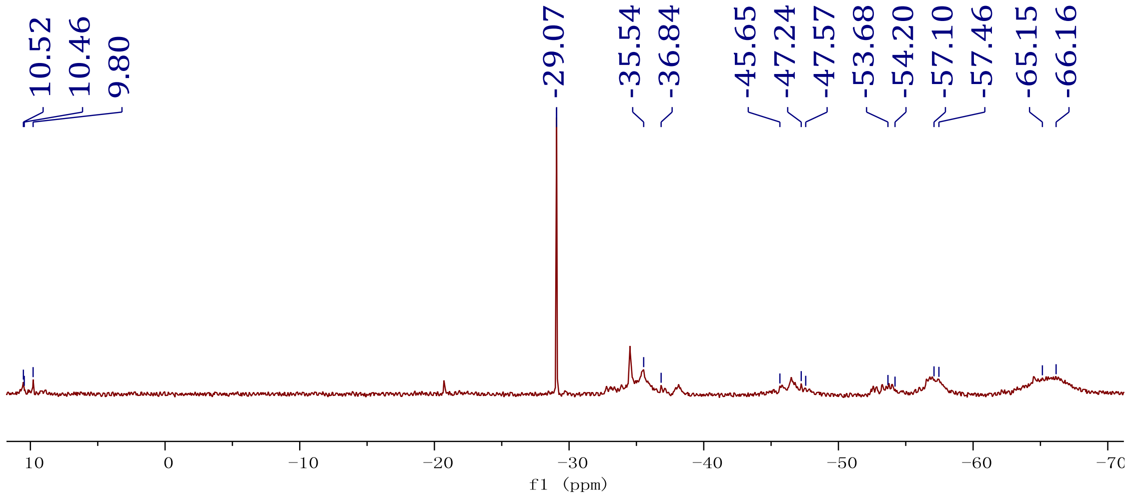

Figure S30. *^{29}Si NMR of D11*

Experimental Design, Materials and Methods

Gel permeation chromatography (GPC) was carried out on a Waters 1525/2414 chromatograph (WATERS Co., America) in a linear column eluted with tetrahydrofuran at a flow rate of 1.0 mL/min. ^{1}H NMR (400MHz, $CDCl_3$, TMS)and ^{29}Si NMR (400MHz, $CDCl_3$) spectra were recorded using a Bruker AVANCE AV 400 MHz spectrometer (Bruker Co., Germany) at room temperature. Thermogravimetric analysis (TGA) was carried out on a TGA 2 thermogravimetric analyzer (Mettler-Toledo AG Co., America) in nitrogen atmosphere from 40 to 700 °C at a heating rate of 10 °C/min.

Acknowledgements

The authors thank the Analysis and Test Center at Zhongkai University of Agriculture and Engineering. The authors also thank everyone at the Guangdong Engineering Research Center of Silicone Electronic Fine Chemicals for their selfless contribution.
